# Structural insights into dimerization and activation of the mGlu2–mGlu3 and mGlu2–mGlu4 heterodimers

**DOI:** 10.1038/s41422-023-00830-2

**Published:** 2023-06-08

**Authors:** Xinwei Wang, Mu Wang, Tuo Xu, Ye Feng, Qiang Shao, Shuo Han, Xiaojing Chu, Yechun Xu, Shuling Lin, Qiang Zhao, Beili Wu

**Affiliations:** 1 State Key Laboratory of Drug Research, Shanghai Institute of Materia Medica, Chinese Academy of Sciences, Shanghai, China; 2 University of Chinese Academy of Sciences, Beijing, China; 3 School of Life Science and Technology, ShanghaiTech University, Shanghai, China; 4 School of Pharmaceutical Science and Technology, Hangzhou Institute for Advanced Study, University of Chinese Academy of Sciences, Hangzhou, Zhejiang China; 5 Zhongshan Institute of Drug Discovery, Shanghai Institute of Materia Medica, Chinese Academy of Sciences, Zhongshan, Guangdong China

**Keywords:** Extracellular signalling molecules, Cryoelectron microscopy

## Abstract

Heterodimerization of the metabotropic glutamate receptors (mGlus) has shown importance in the functional modulation of the receptors and offers potential drug targets for treating central nervous system diseases. However, due to a lack of molecular details of the mGlu heterodimers, understanding of the mechanisms underlying mGlu heterodimerization and activation is limited. Here we report twelve cryo-electron microscopy (cryo-EM) structures of the mGlu2–mGlu3 and mGlu2–mGlu4 heterodimers in different conformational states, including inactive, intermediate inactive, intermediate active and fully active conformations. These structures provide a full picture of conformational rearrangement of mGlu2–mGlu3 upon activation. The Venus flytrap domains undergo a sequential conformational change, while the transmembrane domains exhibit a substantial rearrangement from an inactive, symmetric dimer with diverse dimerization patterns to an active, asymmetric dimer in a conserved dimerization mode. Combined with functional data, these structures reveal that stability of the inactive conformations of the subunits and the subunit–G protein interaction pattern are determinants of asymmetric signal transduction of the heterodimers. Furthermore, a novel binding site for two mGlu4 positive allosteric modulators was observed in the asymmetric dimer interfaces of the mGlu2–mGlu4 heterodimer and mGlu4 homodimer, and may serve as a drug recognition site. These findings greatly extend our knowledge about signal transduction of the mGlus.

## Introduction

The metabotropic glutamate receptors (mGlus) play critical roles in modulating synaptic transmission and neuronal excitability, and dimerization is mandatory for these receptors to exert their functions.^1^ In addition to homodimers, these class C G protein-coupled receptors (GPCRs) were shown to form heterodimers as well.^2–4^ Further evidence supports native existence of the heterodimeric mGlus, such as mGlu2–mGlu4,^3,5,6^ mGlu2–mGlu3,^7^ mGlu2–mGlu7,^8^ and mGlu1–mGlu5,^9^ and suggests their clinical potentials in treating central nervous system (CNS) diseases.^10–12^ The mGlus are divided into three groups; it has been implied that the heterodimers can be formed either within group I (mGlu1, 5), or between and within group II (mGlu2, 3) and group III (mGlu4, 6, 7, 8).^4^ These heterodimeric receptors transduce signals in an asymmetric manner with one specific subunit exclusively mediating G protein activation.^13^ These findings highlight the physiological importance of the mGlu heterodimers and indicate a complex landscape of their functional modulation mechanisms. However, despite recent structure determinations of several mGlu homodimers,^14–19^ molecular details that govern dimerization and activation of the mGlu heterodimers are largely unknown. This limits our understanding about the mGlu signal transduction and modulation, and would hamper future drug development targeting the heterodimeric mGlus. Thus, we performed extensive studies on the mGlu heterodimers, resulting in determination of twelve cryo-electron microscopy (cryo-EM) structures of mGlu2–mGlu3 and mGlu2–mGlu4 in distinct conformational states. Together with functional data, these structures offer important insights into dimer assembly and asymmetric signaling of the mGlu heterodimers.

## Results and discussion

### Structure determination of the mGlu heterodimers

**Fig. 1:**
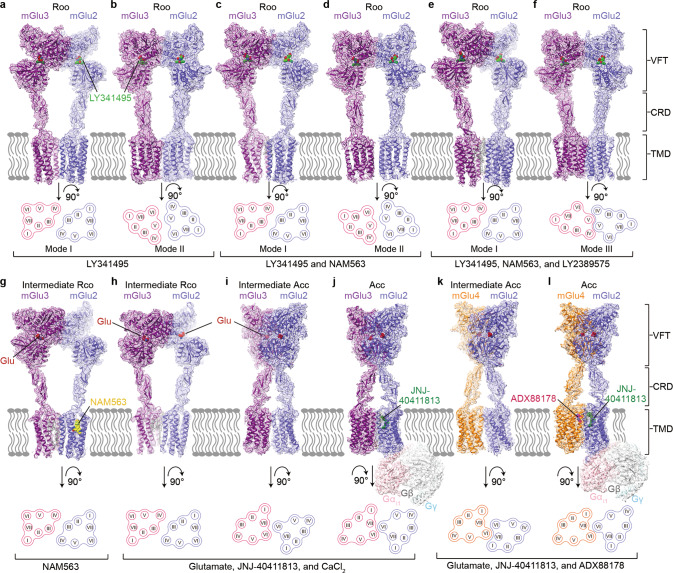
Cryo-EM maps and overall structures of the mGlu2–mGlu3 and mGlu2–mGlu4 heterodimers. The maps and structures are colored according to chains. The mGlu2, mGlu3, and mGlu4 subunits are colored blue, purple, and orange, respectively. Schematic diagrams showing the extracellular view of the TMDs are shown below. The conformational states are labeled on the top, and the ligands used during protein preparation are listed at the bottom. **a**–**f** The antagonist-bound mGlu2–mGlu3. The antagonist LY341495 is shown as spheres with green carbons. The lipid molecules are shown as gray sticks. **a**, **b** The mGlu2–mGlu3 heterodimer in the presence of LY341495 in dimerization modes I (**a**) and II (**b**). **c**, **d** The mGlu2–mGlu3 heterodimer in the presence of LY341495 and NAM563 in dimerization modes I (**c**) and II (**d**). **e**, **f** The mGlu2–mGlu3 heterodimer in the presence of LY341495, NAM563, and LY2389575 in dimerization modes I (**e**) and III (**f**). **g**–**l** The agonist-bound mGlu2–mGlu3 and mGlu2–mGlu4. The agonist glutamate (Glu) is shown as spheres with red carbons. **g** The mGlu2–mGlu3 heterodimer in the presence of NAM563 in the intermediate Rco state. **h**, **i** The mGlu2–mGlu3 heterodimer in the presence of glutamate, JNJ-40411813, and CaCl_2_ in the intermediate Rco (**h**) and intermediate Acc (**i**) states. **j** The G_i1_-bound mGlu2–mGlu3 heterodimer. JNJ-40411813 is shown as spheres with dark green carbons. **k** The G protein-free mGlu2–mGlu4 heterodimer in the presence of glutamate, JNJ-40411813, and ADX88178. **l** The G_i1_-bound mGlu2–mGlu4 heterodimer. ADX88178 is shown as spheres with magenta carbons.

To enable structural studies of the mGlu heterodimers, an FK506-binding protein (FKBP) and a rapamycin-binding fragment (FRB) were linked to the C-termini of the full-length mGlu2 and mGlu3 (or mGlu4), respectively, aiming to stabilize the heterodimerization^20^ (Supplementary information, Fig. S1). The mGlu2-FKBP fusion protein was co-expressed and co-purified with the mGlu3-FRB (or mGlu4-FRB) fusion protein in the presence of different ligands, leading to structure determination of the mGlu heterodimers in different conformational states (Fig. 1; Supplementary information, Figs. S2–S4 and Table S1). To deepen our understanding about behaviors of orthosteric antagonists and negative allosteric modulators (NAMs) in modulating heterodimer conformation, distinct combinations of the group II mGlu antagonist LY341495,^21^ the mGlu2-selective NAM NAM563,^22^ and the mGlu3-selective NAM LY2389575^23^ were used to prepare the mGlu2–mGlu3 heterodimer samples, including (i) LY341495 only, (ii) LY341495 and NAM563, (iii) LY341495, NAM563 and LY2389575, and (iv) NAM563 only, which yielded seven cryo-EM structures in four different states with overall resolutions at 2.8–3.4 Å (Fig. 1a–g; Supplementary information, Figs. S2 and S4a–d, f).

With addition of the agonist glutamate, the mGlu2-selective positive allosteric modulator (PAM) JNJ-40411813,^24^ and CaCl_2_, in which both Ca^2+^ and Cl^–^ are modulators of mGlu3 activation,^25–27^ two structures of the mGlu2–mGlu3 heterodimer at resolutions of 2.8 Å and 3.7 Å were solved, displaying distinct conformations at both the extracellular domains (ECDs) and transmembrane domains (TMDs) (Fig. 1h, i; Supplementary information, Figs. S3a and S4e). To fully activate the heterodimer, the mGlu2–mGlu3 protein was co-purified with the heterotrimeric G_i1_ protein in the presence of glutamate, JNJ-40411813, and CaCl_2_, resulting in a map of the G_i1_-bound mGlu2–mGlu3 heterodimer at 3.3 Å resolution (Fig. 1j; Supplementary information, Figs. S3b and S4g). For mGlu2–mGlu4, the G_i1_-bound complex was prepared by using ADX88178, an mGlu4-selective PAM,^28^ together with glutamate and JNJ-40411813, leading to determination of the G_i1_-bound mGlu2–mGlu4 structure (2.9 Å at the ECDs; 3.4 Å at the TMDs and G_i1_) and a G protein-free structure of the heterodimer (overall 2.9 Å) (Fig. 1k, l; Supplementary information, Figs. S3c and S4h).

Strong densities were observed for the orthosteric ligands in all the structures, the mGlu2 NAM NAM563 in the mGlu2–mGlu3 structure determined in the presence of NAM563 only, and the PAMs in the G_i1_-bound structures, enabling unambiguous modeling of these ligands (Supplementary information, Figs. S5 and S6).

### Diverse dimerization modes of the inactive mGlu2–mGlu3 heterodimer

Previous structural and functional analyses of the mGlu homodimers demonstrate a ligand-dependent conformational rearrangement of the Venus flytrap domains (VFTs) and its importance in triggering receptor activation.^14,16,25^ Upon binding to the orthosteric antagonist, the two VFTs in the mGlu2–mGlu3 heterodimers adopt a similar open conformation with a common dimer interface formed by the top lobes (Supplementary information, Fig. S7a). Nevertheless, relative positions of the bottom lobes are different in the six antagonist-bound structures, which are likely associated with distinct dimerization modes of their TMDs (Supplementary information, Fig. S7a). A difference of the relative orientation of the two VFTs was also observed when comparing the inactive mGlu2–mGlu3 structures with our previously determined structure of the inactive mGlu2–mGlu7^16^ (Supplementary information, Fig. S7a), indicating conformational diversity of the mGlu heterodimers.

**Fig. 2:**
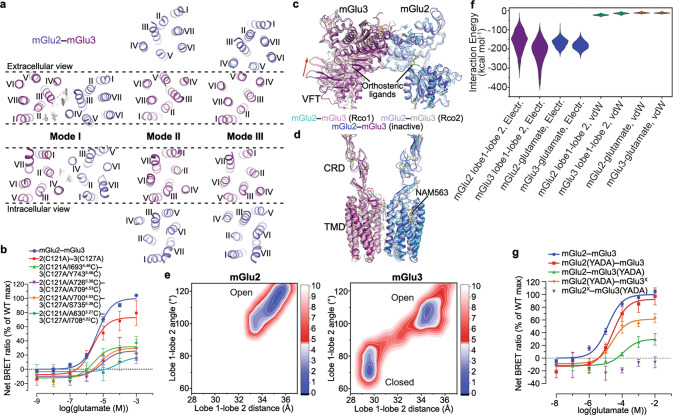
Inactive dimerization modes and Rco conformations of mGlu2–mGlu3. **a** Comparison of the dimerization modes in the inactive mGlu2–mGlu3. The TMDs of the inactive mGlu2–mGlu3 in dimerization modes I, II, and III are shown in both extracellular (top) and intracellular (bottom) views. The mGlu2 and mGlu3 subunits are colored blue and purple, respectively. The lipids in dimerization mode I are shown as gray sticks. The dashed lines indicate that the mGlu3 subunits in the structures are in the same orientation. **b**, **g** Glutamate-induced G_i_ activation of the wild type and mutants of mGlu2–mGlu3 measured by the BRET assay. The BRET data are means ± SEM from at least three independent experiments performed in duplicate. Supplementary information, Table S2 provides detailed independent experiment numbers (*n*), statistical evaluation, and protein expression levels. **c**, **d** Comparison between the Rco conformations and the inactive conformation in dimerization mode I. The Rco structures of mGlu2–mGlu3 in the presence of NAM563 (Rco1) or glutamate, JNJ-40411813, and CaCl_2_ (Rco2) and the inactive structure of mGlu2–mGlu3 in the presence of LY341495, NAM563, and LY2389575 in dimerization mode I are shown at the VFTs (**c**) and the CRDs and TMDs (**d**). The red arrow in **c** indicates the open-to-closed conformational change of the mGlu3 VFT in the Rco structures relative to the inactive structure. The ligands bound in these structures are shown as sticks. **e** 2D free energy landscapes (FELs) spanned by the distance between the centroids of the two lobes and the lobe 1–lobe 2 subdomain angle of mGlu2 and mGlu3. The contours in the 2D subspace are spaced at intervals of 1.0 kcal/mol. **f** The ranges of various interaction energies in the open state, including the electrostatic interaction energies between the lobe 1 and lobe 2 subdomains of mGlu2 and mGlu3 (purple), and between the glutamate molecule and mGlu2 or mGlu3 (blue), as well as the vdW energies between the lobe 1 and lobe 2 subdomains of mGlu2 and mGlu3 (green), and between the glutamate molecule and mGlu2 or mGlu3 (brown), respectively.

Each of the three cryo-EM datasets of mGlu2–mGlu3 in the presence of the antagonist LY341495 yielded two inactive structures with distinct TMD dimerization patterns (Fig. 1a–f; Supplementary information, Fig. S2a–c). Among each pair of the structures, a common TMD dimer interface, which involves the extracellular regions of helices III and IV and the intracellular regions of helices II and IV in the two subunits (termed as dimerization mode I), was observed (Figs. 1a, c, e and 2a). Lacking direct contacts between the helices, the TMDs are mainly associated through lipids. Despite relatively low resolutions in the TMD regions, the cryo-EM maps, except for the map that was obtained in the presence of LY341495 alone, display strong densities for several cholesterol molecules that glue the two subunits together by forming extensive hydrophobic interactions with helices II, III, and IV of mGlu2 and mGlu3 (Fig. 2a; Supplementary information, Fig. S5c, e). In the map of mGlu2–mGlu3 bound to LY341495 only, some densities were observed in the same sites, but did not allow unambiguous modeling of cholesterols due to poor resolution. The importance of the lipid-mediated interactions in stabilizing the inactive state was supported by our mutagenesis studies, in which replacing some key residues adjacent to the cholesterols with alanine or tryptophan increased basal activity of the heterodimer by 30%–70% (Supplementary information, Table S2).

In addition to the lipid-mediated dimerization, two other dimerization patterns of the TMDs were observed in the inactive mGlu2–mGlu3 structures. When bound to the antagonist only or the antagonist together with the mGlu2 NAM, the TMD association of the heterodimer is also mediated by helix V of the subunits (termed as dimerization mode II) (Figs. 1b, d and 2a). Unlike the previously determined structures of the inactive mGlu homo- and heterodimers, in which the two TMDs are completely separated with helix V facing each other,^14,16,18,19,29^ the inactive mGlu2–mGlu3 displays a much closer distance between the subunits with the intracellular regions of helix V and the intracellular tips of helix III forming close contacts (Fig. 2a). However, this dimerization mode was not observed when the mGlu3 NAM LY2389575 was added in addition to the antagonist and mGlu2 NAM. Alternatively, the TMD of mGlu2 undergoes a substantial clockwise rotation (extracellular view), resulting in a tight dimeric association between helices IV and V on the extracellular side and between helices III, IV, and V on the intracellular side (termed as dimerization mode III) (Figs. 1f and 2a). Extra rounds of cryo-EM data processing excluded the existence of mode II in the heterodimers bound to the antagonist and both NAMs, as well as mode III in the heterodimer in complex with the antagonist only or the antagonist together with the mGlu2 NAM. This implies a NAM-induced conformational rearrangement of the TMD region in the heterodimer.

These dimerization modes are confirmed by cysteine crosslinking studies. In contrast to the heterodimer mutant that lacks the inter-VFT disulfide bond (mGlu2(C121A)–mGlu3(C127A)), additional cysteine substitutions of the two residues at the extracellular tips of helices III and IV in the two subunits, respectively (dimerization mode I), substantially increased the dimer proportion (Supplementary information, Fig. S8a). An increased proportion of dimer was also observed when a disulfide bridge was introduced between the intracellular regions of helix V in the subunits (dimerization mode II) or between the extracellular regions of helices IV and V (dimerization mode III) (Supplementary information, Fig. S8a). In a bioluminescence resonance energy transfer (BRET) assay that measures glutamate-induced G_i_ activation, the crosslinked mutants displayed a notably reduced maximal response (*E*_max_) compared to that of the wild-type heterodimer (Fig. 2b; Supplementary information, Table S2), indicating that locking the dimer interface in the inactive states impairs receptor activation.

### Intermediate state of mGlu2–mGlu3 heterodimer

It has been proposed that in addition to the inactive Roo state (‘R’ refers to resting and ‘o’ denotes the open conformation of each VFT) and active Acc state (‘A’ refers to active and ‘c’ denotes the closed conformation of each VFT), intermediate conformational states, such as Rco, Rcc, Aoo, and Aco, may exist in the mGlus.^19,25,30,31^ This underlines the complexity of these dimeric receptors in modulating their functions. However, due to a lack of structural information of the mGlu heterodimers in intermediate states, the molecular mechanism underlying this modulation remains elusive.

In our structural studies of the mGlu2–mGlu3 heterodimer, an intermediate conformation was observed when only the mGlu2 NAM NAM563 was applied to protein preparation. Within the heterodimer, the VFT in mGlu3 exhibits a closed conformation with a glutamate molecule, which most likely comes from the cell culture medium, bound between the two lobes, but the mGlu2 subunit remains in the Apo state with an open conformation at the VFT and has the NAM bound to its TMD (Figs. 1g and 2c, d). The two VFTs dimerize through an interface between the top lobes similar to that in the antagonist-bound structures (Fig. 2c). The cysteine-rich domains (CRDs) adopt separated positions with a distance of 47 Å measured at the Cα atoms of C518 (mGlu2) and C527 (mGlu3), which is also similar to that in the inactive structures, and the dimeric association of the TMDs aligns with the dimerization mode I of the antagonist-bound mGlu2–mGlu3 (Fig. 2d). These structural features indicate that the heterodimer is in an Rco conformational state. The differential behaviors of the two VFTs in agonist coupling likely correlate with the fact that the glutamate affinity of mGlu3 is ~10-fold higher than that of mGlu2,^25,32^ which allows the mGlu3 subunit to bind the agonist first.

An Rco conformation was also observed in one of the two mGlu2–mGlu3 structures in the presence of glutamate, CaCl_2_, and the mGlu2 PAM JNJ-40411813. This structure exhibits an overall architecture similar to that of the above NAM-bound Rco structure (Fig. 2c, d). However, the open VFT of the mGlu2 subunit is also occupied with a glutamate molecule, which attaches to the top lobe (Figs. 1h and 2c). This finding suggests that glutamate triggers the conformational rearrangement of the VFT by initially recognizing the binding site in the top lobe, which is rich in basic residues, and subsequently inducing a relative movement of the two lobes, resulting in the closure of the domain.

The two Rco structures of mGlu2–mGlu3 imply a sequential conformational rearrangement of the VFTs. This was further studied by all-atom molecular dynamics (MD) simulations with an enhanced sampling technique called Gaussian accelerated molecular dynamics (GaMD).^33,34^ The simulations were performed on a model of the VFT heterodimer of mGlu2–mGlu3 with both subunits adopting the open conformation and a glutamate molecule binding to each top lobe. Through four independent trajectories with an accumulated simulation time of ~5 µs, the mGlu3 VFT accomplished a conformational change from the open state to closed state, while the mGlu2 VFT mainly maintained its open conformation, as shown by the distance between the centroids of the two lobes (top lobe, lobe 1; bottom lobe, lobe 2) and the lobe 1–lobe 2 angle along the trajectories (Supplementary information, Fig. S7b) as well as free energy landscapes generated with such distance and angle (Fig. 2e). Therefore, the two VFT lobes of mGlu3 are apparently more preferential to close up. We further analyzed the electrostatic interaction between the two lobes, and a lower electrostatic interaction energy was found between the lobes of mGlu3 than those for mGlu2 in their open states (Fig. 2f). In addition, the decrease in the electrostatic interaction energy is much more substantial than that in the van der Waals (vdW) energy along the conformational change pathway of mGlu3 (Supplementary information, Fig. S7c). It is thus speculated that the electrostatic interactions act as a driving force for the domain motion of the mGlu2–mGlu3 VFTs, while the stronger electrostatic interactions between the two lobes in mGlu3 enable the easier occurrence of the open-to-closed conformational change as compared to mGlu2.

To further understand the role of the VFT conformational change in modulating heterodimer activation, we introduced the ‘glutamate-insensitive’ mutations YADA^35^ in the glutamate-binding site of either subunit in mGlu2–mGlu3 (mGlu2, Y216A and D295A; mGlu3, Y222A and D301A), and measured the glutamate-induced G_i_ activation. The data showed a 10-fold decrease of the glutamate potency (EC_50_) and a 60% reduction of *E*_max_ when the mutations were introduced in the mGlu3 subunit, but a wild-type level of activity if the mGlu2 subunit carried the mutations (Fig. 2g; Supplementary information, Table S2). Given that the two mutations are within the bottom lobe of the VFT, it is likely that the mutated subunit can still bind the agonist with the top lobe but its closure is hampered. Thus, the BRET data suggest that closure of the mGlu3 VFT, but not the mGlu2 VFT, is sufficient for full activation of the heterodimer. This is in contrast to the previous findings that closure of both VFTs is required for maximal activation of the mGlu homodimers^35^ and mGlu2–mGlu4 heterodimer.^6^ However, this does not rule out the possibility that the closure of one subunit induces a spontaneous closure of its neighboring subunit. If this is the case, it is apparent that mGlu2 is not as efficient as mGlu3 in triggering the closure of its heteromeric partner, allowing only a proportion of the heterodimers to reach the active Acc state. Taken together, our structural and functional data highlight that the two VFTs modulate the activation of mGlu2–mGlu3 heterodimer in an asymmetric manner, and strongly imply that the VFT of mGlu3 plays a dominant role in governing the heterodimer signaling.

### Asymmetric activation of the mGlu heterodimers

**Fig. 3:**
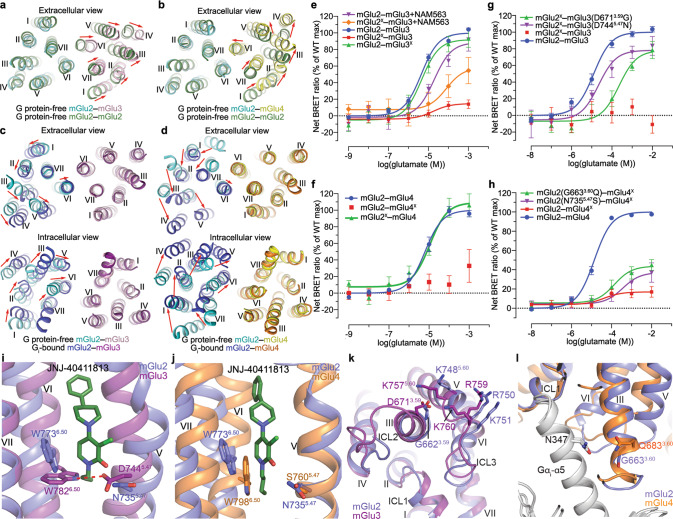
Asymmetric dimerization and activation of mGlu2–mGlu3 and mGlu2–mGlu4. **a**, **b** Comparison of the TMDs in the G protein-free, agonist-bound structures of the heterodimers and mGlu2 homodimer (PDB ID: 7EPB). mGlu2–mGlu3 and mGlu2 (**a**); mGlu2–mGlu4 and mGlu2 (**b**). The structures are aligned at the mGlu2 subunit of the heterodimer, and shown in an extracellular view. The red arrows indicate the movement of each helix in the mGlu3 subunit (**a**) or mGlu4 subunit (**b**) of the heterodimer relative to its counterpart in the homodimer structure. **c**, **d** Comparison of the TMDs in the G protein-free and -bound structures of the heterodimers. mGlu2–mGlu3 (**c**); mGlu2–mGlu4 (**d**). The structures are aligned at the G protein-free subunits, and shown in both extracellular and intracellular views. The red arrows indicate the movement of each helix in the G_i_-bound mGlu2 subunit relative to its counterpart in the G protein-free structure. **e**–**h** Glutamate-induced G_i_ activation of the heterodimers measured by the BRET assay. mGlu2–mGlu3 and mutants (**e**, **g**); mGlu2–mGlu4 and mutants (**f**, **h**). The superscript ‘X’ indicates that the G protein coupling of the subunit was blocked by introducing a mutation in ICL3 (mGlu2, F756S; mGlu3, F765S; mGlu4, F781S). The BRET data are means ± SEM from at least four independent experiments performed in duplicate. Supplementary information, Table S2 provides detailed independent experiment numbers (*n*), statistical evaluation, and protein expression levels. **i**, **j** Comparison of the conformations of the residue W^6.50^ in the two subunits in the G_i_-bound heterodimer structures. mGlu2–mGlu3 (**i**); mGlu2–mGlu4 (**j**). The residues at positions 5.47 and 6.50 in both subunits are shown as sticks. The PAM JNJ-40411813 bound to the mGlu2 subunit, which stabilizes the active conformation of the residue W773^6.50^, is shown as green sticks. The hydrogen bond between the residues D744^5.47^ and W782^6.50^ in the mGlu3 subunit is indicated by a red dashed line (**i**). **k** Comparison of the interactions between the residue 3.59 and the basic residues in helix V and ICL3 of mGlu2 and mGlu3. Due to lack of densities for the residue D671^3.59^ in the mGlu2–mGlu3 structures and previously determined mGlu3 homodimer structures, we generated a model of the mGlu3 TMD by SWISS-MODEL server using the mGlu2 TMD structure in the G_i1_–mGlu2–mGlu3 complex as a template. The mGlu3 TMD model and mGlu2 TMD structure (PDB ID: 7EPE) are shown in cartoon representation. **l** Comparison of the interaction between the mGlu residue 3.60 and the Gα_i_ residue N347 in mGlu2 and mGlu4. The G_i_-bound subunits and Gα_i_ subunits in the G_i_-bound structures of mGlu2 and mGlu4 homodimers are shown in cartoon representation.

Upon activation, the mGlu2–mGlu3 and mGlu2–mGlu4 heterodimers undergo a notable conformational rearrangement. Closure of the VFTs brings about close proximity of the CRDs and an alteration of the TMD dimerization mode (Fig. 1i–l). In contrast to the conformational deviation of the VFTs in the inactive structures, the agonist-bound VFTs exhibit an identical closed conformation in both the G protein-bound and -free structures of the mGlu2–mGlu3 and mGlu2–mGlu4 heterodimers, which resembles the VFT conformation observed in the agonist-bound structures previously determined for their parent homodimers (Supplementary information, Fig. S7d). In the absence of G protein, the agonist-bound heterodimers display an asymmetric dimer interface in the extracellular regions of the TMDs (Fig. 3a, b). This is in contrast to the mGlu homodimers, of which the TMDs form a symmetric dimer along the whole length of helix VI.^14,16,18,19^ Compared to the G protein-free structure of the mGlu2 homodimer, the TMD of mGlu3 in mGlu2–mGlu3 undergoes a shift away from the mGlu2 subunit, leading to a dimer interface mediated by helix VI in mGlu2 and helices VI and VII in mGlu3 (Fig. 3a). For mGlu2–mGlu4, the mGlu4 subunit rotates anticlockwise by ~20° (extracellular view) with its helix VI forming extensive interactions with helices I, VI, and VII of mGlu2 (Fig. 3b). This finding demonstrates the asymmetry of the two subunits in mGlu heterodimerization and highlights differential behaviors of one certain mGlu subunit in governing assembly of different heterodimers.

It has been suggested that the mGlu heterodimers transduce signals in an asymmetric mode of action with one designated subunit coupling to G protein.^13^ The asymmetric dimerization patterns in the G protein-free, agonist-bound structures of the heterodimers, of which the dimer association is mediated by helix VI in one subunit and helices I, VI, and VII in the other subunit, are similar to those observed in the previously determined G_i_-bound structures of the mGlu2 and mGlu4 homodimers, where helix VI in the G_i_-bound subunit and helices I, VI, and VII of the G_i_-free subunit mediate dimerization.^15^ This similarity raises a question about whether the heterodimerization pattern in the G protein-free state correlates with the asymmetric signal transduction of the heterodimer. Indeed, the mGlu2 subunit, of which helix VI mediates heterodimerization in the G protein-free mGlu2–mGlu3 structure, couples to the G_i1_ protein in the G_i1_-bound mGlu2–mGlu3 structure through a similar interaction pattern to those in the G_i_-bound mGlu homodimers (Supplementary information, Fig. S7e). Compared to the G protein-free mGlu2–mGlu3, the mGlu2 TMD in the G_i1_-bound structure shifts towards the mGlu3 subunit, generating more extensive interactions between helix VI of mGlu2 and helices I, VI, and VII of mGlu3 (Fig. 3c).

The role of the mGlu2 subunit in G protein activation in mGlu2–mGlu3 is supported by functional assays using a quality control system that only allows the heterodimer to reach cell surface.^13^ The results showed that the heterodimer activated the G_i_ protein at a wild-type level, when the F765S substitution that blocks G protein binding was introduced in the third intracellular loop (ICL3) of the mGlu3 subunit (mGlu2–mGlu3^X^); however, a considerable reduction of G_i_ activation was observed if mGlu2 carried the corresponding mutation (mGlu2^X^–mGlu3) (Fig. 3e; Supplementary information, Table S2). We further investigated the asymmetric signal transduction of mGlu2–mGlu3 by combining the VFT mutations YADA with the G protein-coupling-deficient mutation in ICL3. It was shown that the mGlu2^X^–mGlu3(YADA) mutant lost the ability to activate the G_i_ protein, whereas the mGlu2(YADA)–mGlu3^X^ mutant only displayed a limited reduction of *E*_max_ (Fig. 2g; Supplementary information, Table S2). These data demonstrate that closure of the mGlu3 VFT is sufficient to activate the mGlu2 TMD. This *trans*-activation pattern of the mGlu heterodimer is similar to the signal transduction mode of the GABA_B_ receptor, where the GB1 subunit is responsible for agonist binding and the GB2 subunit couples to the G protein.^36^

For the mGlu2–mGlu4 heterodimer, the dimerization mode in the G protein-free state suggests that mGlu4 is the subunit that transduces signals. This is supported by the previous study reporting that the group III subunits are responsible for G protein activation in the heterodimers between the group II and III mGlus,^13^ and was further confirmed by our functional data showing that the mGlu2-blocked heterodimer (mGlu2^X^–mGlu4) was activated by glutamate at a similar level to that of the wild-type heterodimer while no response was observed for the mGlu2–mGlu4^X^ heterodimer (Fig. 3f; Supplementary information, Table S2). However, to our surprise, the G_i1_ protein binds to the mGlu2 subunit in the G_i1_-bound mGlu2–mGlu4 structure (Fig. 1l). To allow the G protein binding, the helical bundle of mGlu2 undergoes a notable anticlockwise rotation (extracellular view), which switches the dimer interface from helices I/VI/VII (mGlu2)–helix VI (mGlu4) to helix VI (mGlu2)–helices I/VI/VII (mGlu4) (Fig. 3d). The G protein binding to the mGlu2 subunit in mGlu2–mGlu4 most likely results from distinct binding modes and modulation mechanisms of the mGlu2 and mGlu4 PAMs that co-bind to the heterodimer, which will be discussed later (see the next section).

How the mGlu heterodimers choose the subunit for G protein activation is central to understanding the asymmetric signal transduction of these dimeric receptors, but the molecular mechanism remains unknown. It was reported that in the mGlu2–mGlu4 heterodimer, G protein activation by mGlu2 occurred if the mGlu4 TMD was inhibited by a NAM.^13^ A similar result was also obtained for mGlu2–mGlu3, in which the mGlu2-selective NAM NAM563 partially rescued the G_i_ activation of mGlu2^X^–mGlu3 by stabilizing the mGlu2 TMD in the inactive state (Fig. 3e; Supplementary information, Table S2). These data raise a possibility that the stability of the inactive conformation of a subunit determines its contribution to G protein activation in the heterodimers.

The highly conserved class C GPCR residue W^6.50^ (superscript refers to a modified form of the Ballesteros-Weinstein numbering system for class C GPCRs), which displays a rotamer conformational change when comparing the inactive and active structures of mGlu2, has been suggested as a ‘micro-switch’ that is the key to mGlu activation.^15,16^ This was also observed in the G_i1_-bound mGlu2–mGlu3 structure, where the W^6.50^ residues in the G_i1_-bound mGlu2 and G protein-free mGlu3 adopt different rotamer conformations (Fig. 3i). In the mGlu3 subunit, side chain of the acidic residue D744^5.47^ forms a hydrogen bond with the side-chain nitrogen of W782^6.50^, potentially stabilizing the bulky residue in the inactive state. However, in mGlu2, this residue is replaced by N735^5.47^, which is a weaker hydrogen acceptor compared to the negatively charged aspartate residue in mGlu3. Thus, the corresponding hydrogen bond in mGlu2 is weaker and less effective in constraining the rotamer conformational change of W^6.50^. This difference results in a more stable inactive conformation of mGlu3 compared to mGlu2, providing a potential structural basis for the mGlu2–mGlu3 heterodimer signaling through the mGlu2 subunit. This is supported by our mutagenesis studies, in which the mGlu2-blocked heterodimer mGlu2^X^–mGlu3 gained signaling when the D744^5.47^N mutation was introduced in mGlu3 (Fig. 3g; Supplementary information, Table S2), indicating that the ability of the mGlu3 subunit to mediate G protein activation increases once its inactive state is destabilized. Furthermore, on the intracellular surface of mGlu3, the residue D671^3.59^ potentially forms ionic interactions with three basic residues K757^5.60^, R759, and K760 in helix V and ICL3, which may stabilize the inactive conformation of the intracellular region, while this is absent in mGlu2 as the acidic residue is substituted by a glycine (Fig. 3k; Supplementary information, Fig. S8b). The importance of these interactions in inhibiting mGlu3 activation was also confirmed by our mutagenesis data showing that the D671^3.59^G substitution of mGlu3 substantially rescued G_i_ activation of mGlu2^X^–mGlu3 (Fig. 3g; Supplementary information, Table S2).

The notion that stability of the inactive conformations of the subunits determines subunit preference for G protein activation was also verified in the mGlu2–mGlu4 heterodimer. In mGlu4, the residue at the key position 5.47 is serine with a short side chain, which is incapable of forming interaction with W^6.50^ and loses the stabilizing effect on the rotamer conformational change. Indeed, the W^6.50^ residues in the G protein-free mGlu4 subunits of the G_i1_–mGlu2–mGlu4 and mGlu4–G_i3_ complexes display a rotamer conformation similar to that in the active subunits (Fig. 3j). This likely results in a more unstable inactive conformation of mGlu4 in comparison with mGlu2, which facilitates signal transduction through this subunit in the heterodimer. Indeed, the N735^5.47^S substitution in the mGlu2 subunit elevated the *E*_max_ of glutamate-induced G_i_ activation of mGlu2–mGlu4^X^ (Fig. 3h; Supplementary information, Table S2). The residue S^5.47^ is highly conserved in group III mGlus (Supplementary information, Fig. S8b), and this may explain the previous observation that the group III subunits mediate signaling in the heterodimers between group II and III mGlus.^13^ In addition, in the G protein-binding interface, the mGlu4 residue Q683^3.60^ (also conserved in group III mGlus; Supplementary information, Fig. S8b) engages in a hydrogen bond with the residue N347 in the α5 helix of the Gα_i_ subunit as shown in the mGlu4–G_i3_ structure, which is not present in mGlu2 due to a glycine replacement at this position (G663^3.60^) (Fig. 3l). This interaction strengthens the receptor–G protein interaction and may increase the ability of mGlu4 to transduce signals in the heterodimer. This is supported by our functional data showing that the mGlu2 mutation G663^3.60^Q partially rescued the signaling of mGlu2–mGlu4^X^ (Fig. 3h; Supplementary information, Table S2). Taken together, our structural and functional data imply that some key residues that govern the stability of the inactive conformation and G protein coupling of the subunits are determinants of the asymmetric signal transduction of the mGlu heterodimers.

### Distinct modulation mechanisms of the mGlu2 and mGlu4 PAMs

It has long been acknowledged that the mGlu PAMs have a major role in modulating signaling of the mGlu homo- and heterodimers through interactions with the receptor TMD.^13,37^ Indeed, the mGlu2 PAM JNJ-40411813 or the mGlu4 PAM ADX88178 alone can activate the mGlu2–mGlu4 heterodimer (Supplementary information, Fig. S8c, d and Table S2), demonstrating that a single PAM is sufficient to activate the heterodimer.

**Fig. 4:**
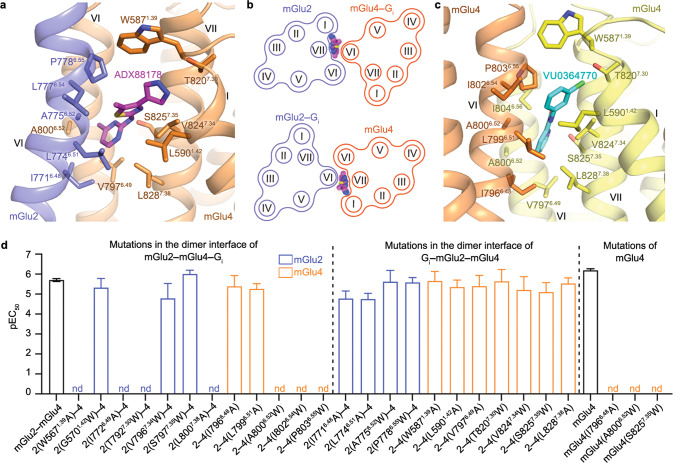
PAM binding modes. **a** Binding mode of ADX88178 in the mGlu2–mGlu4 heterodimer. The G_i_-bound structure of mGlu2–mGlu4 is shown in cartoon representation, with mGlu2 and mGlu4 colored blue and orange, respectively. The PAM ADX88178 is shown as magenta sticks. The residues in the two subunits that interact with ADX88178 are shown as sticks. **b** Schematic diagrams showing that ADX88178 can bind to the dimer interface in mGlu2–mGlu4 when either of the subunits couples to the G protein. Top, mGlu2–mGlu4–G_i_; bottom, G_i_–mGlu2–mGlu4. **c** Binding mode of VU0364770 in the mGlu4 homodimer. The mGlu4–G_i3_ structure is shown in cartoon representation, with the G protein-bound subunit and the G protein-free subunit colored orange and yellow, respectively. The PAM VU0364770 is shown as cyan sticks. **d** ADX88178-induced G_i_ activation of mGlu2–mGlu4 heterodimer and mGlu4 homodimer. Bars represent calculated PAM potency (pEC_50_), and are colored according to locations of the mutations. See Supplementary information, Table S2 for values of *E*_max_. The mGlu2–mGlu4 mutations are divided into two groups, one including the mutations in the potential dimer interface of the mGlu2–mGlu4–G_i_ complex and the other including the mutations in the dimer interface of the G_i_–mGlu2–mGlu4 complex. Data are means ± SEM from at least three independent experiments performed in duplicate. Supplementary information, Table S2 provides detailed independent experiment numbers (*n*), statistical evaluation, and protein expression levels.

Similar to what was previously observed in the mGlu2–G_i1_ structure,^15^ the mGlu2 PAM JNJ-40411813 binds to a pocket formed by helices III, V, VI, and VII within the mGlu2 subunit of mGlu2–mGlu4 (Fig. 1j, l). Unexpectedly, the mGlu4 PAM ADX88178 adopts a completely different binding mode. Instead of binding inside the helical bundle, it occupies a binding site in the asymmetric dimer interface in the G_i1_–mGlu2–mGlu4 structure and forms extensive contacts with both subunits mainly through hydrophobic interactions (Figs. 1l and 4a). The methylpyrimidine ring points towards helix VI in mGlu4 and is clamped by helix VI of mGlu2 and helix VII of mGlu4, which form further interactions with the thiazole group of the PAM, while the pyrazol ring mainly makes contacts with helix I of mGlu4. Sequence alignment reveals that most of the residues at this binding site are conserved in mGlu2 and mGlu4, except for one residue (G570^1.42^ in mGlu2; L590^1.42^ in mGlu4) (Supplementary information, Fig. S8b). This suggests that this PAM can bind to the heterodimer interfaces of both helix VI (mGlu2)–helices I/VI/VII (mGlu4) (interface in the G_i1_–mGlu2–mGlu4 complex) and helices I/VI/VII (mGlu2)–helix VI (mGlu4) (potential interface in the mGlu2–mGlu4–G_i1_ complex), given the conserved dimerization pattern wherein the G protein-bound subunit utilizes helix VI to dimerize with helices I, VI, and VII in the G protein-free subunit as mentioned above (Fig. 4b). To verify this binding mode, we performed mutagenesis studies on both interfaces by replacing the key residues involved in ADX88178 binding in the G_i1_–mGlu2–mGlu4 structure and the corresponding residues in their dimeric partners with alanine or tryptophan. Interestingly, although all these mutants had a wild-type level of glutamate-induced G_i_ activation (Supplementary information, Fig. S8g, h), a substantial impairment of ADX88178-induced G_i_ activation was observed for the heterodimers with mutations in the potential dimer interface of mGlu2–mGlu4–G_i1_ (Fig. 4d; Supplementary information, Fig. S8e and Table S2), but the corresponding mutations in the dimer interface of G_i1_–mGlu2–mGlu4 displayed a limited effect (Fig. 4d; Supplementary information, Fig. S8f and Table S2). These data support the PAM binding mode and are consistent with the fact that the activation of mGlu2–mGlu4 is mediated by the mGlu4 subunit in the absence of mGlu2 PAM.

The PAM binding mode observed in the mGlu2–mGlu4 heterodimer suggests that the mGlu4 PAM may also activate the mGlu4 homodimer in the same manner. Indeed, by re-processing our previous cryo-EM data of the mGlu4–G_i3_ complex,^15^ we obtained an improved cryo-EM map (ECDs, 2.9 Å; TMDs–G_i3_, 3.3 Å) (Supplementary information, Fig. S3d), which displays strong densities for the mGlu4 PAM VU0364770^38^ at a similar binding site in the asymmetric dimer interface (Fig. 4c; Supplementary information, Fig. S6f). Mutagenesis studies showed that some mutations within the binding pocket dramatically decreased the G_i_ activation of the homodimer induced by the PAM, ADX88178 or VU0364770, but had little effect on the glutamate-induced activity (Fig. 4d; Supplementary information, Fig. S8i–k and Table S2).

By further studying the signal transduction of mGlu2–mGlu4, we found that in the absence of the orthosteric agonist, JNJ-40411813 alone activated mGlu2–mGlu4^X^ but not mGlu2^X^–mGlu4, whereas ADX88178 produced signals only through mGlu2^X^–mGlu4 (Supplementary information, Fig. S8c, d and Table S2). These data indicate that the mGlu2 and mGlu4 PAMs can activate only one of the subunits, however, their modulation mechanisms are different. The mGlu2 PAM exclusively activates the mGlu2 subunit by inducing a conformational rearrangement of the TMD,^15^ which destabilizes the inactive state of the subunit and thus shifts the signaling pathway from mGlu4 to mGlu2. In contrast, the binding site of ADX88178 in the dimer interface strongly implies that this PAM modulates receptor signaling by stabilizing the asymmetric dimerization that is required for activation of the mGlu dimers. However, binding with such a PAM does not alter the stability of the inactive conformations of the subunits in the heterodimer due to a lack of effect on the intra-subunit conformational change. The different modulation mechanisms of these PAMs agree with our G_i1_–mGlu2–mGlu4 structure in the presence of both JNJ-40411813 and ADX88178, in which the mGlu2 subunit binds to the G protein. Taken together, our structural and functional data reveal distinct modulation mechanisms of different mGlu PAMs and underline the complexity of these modulators in governing the heterodimer functionality.

**Fig. 5:**
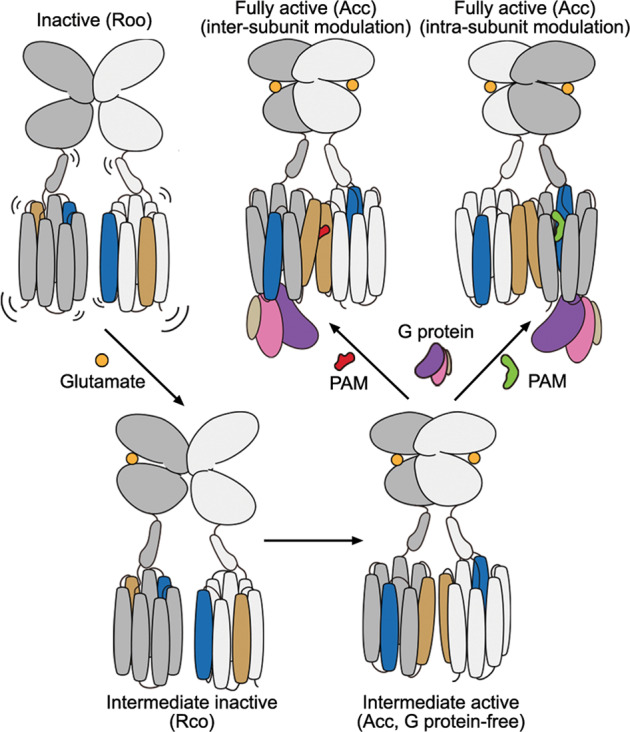
Schematic diagrams summarizing the conformational changes of the mGlu heterodimers during activation. Helices IV and VI in the TMDs are highlighted by colors of blue and brown, respectively. The PAMs that modulate the heterodimer activity in inter- and intra-subunit manners are colored red and green, respectively.

In summary, this work provides new insights into dimerization, signal transduction and function modulation of the mGlu heterodimers. The signal transduction is initiated from a sequential conformational change of the VFTs to a rearrangement of the TMDs that switch from an inactive, symmetric dimer with multiple dimerization modes to an active, asymmetric dimer with a conserved dimerization pattern (Fig. 5). Multiple intermediate conformations, including the intermediate Rco and Acc (G protein-free) conformations as well as other possible conformations, facilitate the conformational rearrangement. The asymmetric signaling is further reflected by different abilities of the two subunits in G protein coupling, which are likely determined by the stability of their inactive conformations and the interaction patterns with the G protein, and are modulated by PAMs that exert a positive effect on heterodimer activation in an intra- or inter-subunit manner (Fig. 5). The mechanism underlying the asymmetric signal transduction of the heterodimers is distinct from that of the mGlu homodimers, as the latter is mainly modulated by asymmetric dimerization.^15^ These findings highlight the complexity and diversity of the mGlu signaling mechanisms.

## Materials and methods

### Construct cloning and protein expression

To improve protein expression, the human *GRM2* (Uniprot Q14416), *GRM3* (Uniprot Q14832) and *GRM4* (Uniprot Q14833) genes were cloned into pTT5 vector with the original signal peptide replaced by haemagglutinin (HA) signal peptide followed by a Flag epitope tag (DYKDDDD). Two fusion proteins FKBP and FRB were introduced to the C-termini of mGlu2 and mGlu3 (or mGlu4), respectively, to facilitate heterodimer formation as previously described.^16,20^ The PreScission protease (PPase) site was inserted between the receptor and fusion protein to allow removal of the fusion proteins. To allow tandem affinity chromatography, a 10× His tag was added to the C-terminus of mGlu2-FKBP and a 2× Strep tag was added to the N-termini of mGlu3-FRB and mGlu4-FRB.

The mGlu2 construct was co-expressed with the mGlu3 or mGlu4 construct (plasmid ratio, 1:1; plasmid final concentration, 1 μg/mL) in HEK293F cells (Invitrogen; cells were routinely tested for mycoplasma contamination) with the starting density at 1.5 × 10^6^ cells/mL. Rapamycin (MedChemExpress) was added to a final concentration of 100 nM during expression to facilitate heterodimerization of FKBP and FRB. The cells were cultivated in 5% CO_2_ at 37 °C for 48 h, and then collected by 1000× *g* centrifugation for 15 min and stored at –80 °C until use.

### Purification of mGlu2–mGlu3 and mGlu2–mGlu4 heterodimers

The membranes of HEK293F cells expressing the mGlu2–mGlu3 or mGlu2–mGlu4 heterodimer were lysed by repeated dounce homogenization in a hypotonic buffer containing 10 mM HEPES, pH 7.5, 10 mM MgCl_2_, 20 mM KCl, and EDTA-free protease inhibitor cocktail tablets (Roche) and ultracentrifuged at 160,000× *g* for 30 min to isolate the membrane fractions. The membranes were then resuspended by dounce homogenization in a high-osmotic buffer (hypotonic buffer supplemented with 1 M NaCl) followed by ultracentrifugation at 160,000× *g* for 30 min. The purified membranes were suspended in the hypotonic buffer supplemented with 30% glycerol and stored at –80 °C until use.

The membranes were thawed on ice and incubated with different ligands at 4 °C for 1 h. To stabilize the mGlu2–mGlu3 heterodimer in inactive state, different ligand combinations were used, including (i) 50 μM LY341495 (MedChemExpress), (ii) 50 μM LY341495 and 50 μM NAM563 (provided by Merck), (iii) 50 μM LY341495, 50 μM NAM563, and 50 μM LY2389575 (Tocris), and (iv) 50 μM NAM563. For the intermediate active state of mGlu2–mGlu3, 10 mM glutamate, 50 μM JNJ-40411813 (Tocris), and 5 mM CaCl_2_ were added. The ligands at the same concentrations were added to all buffers in the following procedures of purification.

The heterodimers were extracted from the cell membranes by adding equal volume of buffer containing 50 mM HEPES, pH 7.4, 2% (w/v) *n*-dodecyl-β-d-maltopyranoside (DDM, Anatrace), 0.4% (w/v) cholesteryl hemisuccinate (CHS, Sigma), and 300 mM NaCl, and incubated at 4 °C for 3 h. The supernatant was collected by centrifugation at 160,000× *g* for 30 min and then incubated with TALON resin (Clontech) in the presence of 10 mM imidazole at 4 °C overnight. The TALON resin was then collected by centrifugation at 800× *g* for 5 min, and washed with 100 column volumes (CVs) of buffer A containing 0.05% (w/v) DDM, 0.01% (w/v) CHS, 25 mM HEPES, pH 7.5, 150 mM NaCl, and 30 mM imidazole. The proteins were eluted by 6 CVs of buffer A supplemented with 300 mM imidazole, and further incubated with STREP resin (IBA) at 4 °C overnight. The STREP resin was collected by centrifugation at 800× *g* for 5 min and washed with 20 CVs of buffer A in the absence of 30 mM imidazole. After that, the detergent was exchanged to glycol-diosgenin (GDN, Anatrace) by incubating the resin in a buffer containing 25 mM HEPES, pH 7.4, 0.25% (w/v) GDN, and 150 mM NaCl at 4 °C for 2 h. The STREP resin was further washed with 20 CVs of buffer B that contains 25 mM HEPES, pH 7.4, 0.01% (w/v) GDN, and 150 mM NaCl. The heterodimers were subsequently eluted by 4 CVs of buffer C containing 200 mM Tris, pH 8.0, 0.01% (w/v) GDN, 150 mM NaCl, and 50 mM biotin. The final samples were concentrated to ~10 mg/mL.

To obtain the G_i_-bound heterodimers, 1 mg/mL His-tagged dominant-negative Gα_i1_ (prepared as described below), 60 μg/mL His-tagged PPase (custom-made), 50 μg/mL scFv16^39^ (prepared as described below), and 25 mU/mL Apyrase (New England Biolabs, NEB) were added during the incubation procedure of STREP resin and the protein samples were prepared following the same protocol as mentioned above. 10 mM glutamate, 50 μM JNJ-40411813, and 5 mM CaCl_2_ (for mGlu2–mGlu3) or 30 μM ADX88178 (MedChemExpress) (for mGlu2–mGlu4) were added to all the buffers.

### Expression and purification of G_i1_ protein and scFv16

The dominant-negative heterotrimeric G_i1_ protein was generated as previously described^40^ by introducing the S47N, G203A, E245A, and A326S substitutions in the human Gα_i1_ subunit (DNGα_i1_). The DNGα_i1_, 6× His-tagged human Gβ_1_, and Gγ_2_ subunits were co-expressed in HighFive insect cells (Invitrogen) using the Bac-to-Bac Baculovirus Expression System (Invitrogen). The cells at a density of 1.5 × 10^6^ cells/mL were infected with the baculoviral stocks of DNGα_i1_ and Gβ_1_γ_2_ at a multiplicity of infection (MOI) ratio of 1:1. After 48 h, the cells were collected by centrifugation at 800× *g* for 15 min, lysed by repeated dounce homogenization in lysis buffer containing 10 mM Tris, pH 7.5, 100 μM MgCl_2_, 5 mM β-mercaptoethanol, 50 μM GDP, and protease inhibitor cocktail tablets (Roche), and then solubilized by 1% sodium cholate and 0.05% DDM. After centrifugation at 160,000× *g* for 30 min, the supernatant was incubated with TALON resin in the presence of 5 mM imidazole at 4 °C overnight. The TALON resin was washed with 50 CVs of buffer D containing 20 mM HEPES, pH 7.5, 100 mM NaCl, 0.05% DDM, 5 mM MgCl_2_, 20 μM GDP, and 30 mM imidazole, and then eluted with 4 CVs of buffer D supplemented with 300 mM imidazole. The protein was concentrated to 5 mg/mL and freshly prepared when needed.

The 6× His-tagged scFv16 was generated as previously described^39^ and a PPase site was inserted between scFv16 and the C-terminal 6× His tag. The protein was expressed in HighFive insect cells as a secreted form using the Bac-to-Bac Baculovirus Expression System. High-titer viral stock of scFv16 at an MOI of 5 was used to infect the cells at a density of 1.5 × 10^6^ cells/mL. The supernatant was collected 48 h post infection, pH-balanced by addition of 50 mM Tris, pH 7.5, and supplemented with 1 mM NiCl_2_ and 5 mM CaCl_2_. The mixture was incubated at 25 °C for 1 h, filtered by 0.22-μm filter membrane (Millipore) to discard precipitate, and further incubated with Ni-NTA resin at 4 °C for 1 h. The resin was washed with 20 CVs of high-salt wash buffer containing 20 mM HEPES, pH 7.5, 500 mM NaCl, and 10 mM imidazole followed by 20 CVs of low-salt wash buffer containing 20 mM HEPES, pH 7.5, 100 mM NaCl, and 10 mM imidazole. The protein was eluted by the low-salt wash buffer supplemented with 250 mM imidazole and the eluate was dialyzed into a buffer containing 20 mM HEPES, pH 7.5 and 100 mM NaCl. The C-terminal 6× His tag was removed by cleavage with His-tagged PPase at 4 °C overnight. The sample was further incubated with Ni-NTA resin at 4 °C for 1 h to remove the His-tagged PPase and cleaved 6× His tag. The purified protein was collected and concentrated to 1.5 mg/mL and flash frozen until further use.

### Cryo-EM data acquisition

The cryogenic specimens of the G_i1_-bound heterodimers were prepared at a protein concentration of ~10 mg/mL, while for the rest of samples, the protein concentration was ~8 mg/mL. Three microliters of the purified samples was applied to freshly glow-discharged 300-mesh amorphous NiTi foil 1.2/1.3 (Au)^41^ and the excess sample was blotted away with blot force of 0 and blot time ranging from 1.5 s to 2 s at 4 °C and 100% humidity. The grids were subsequently plunge-frozen in liquid ethane cooled by liquid nitrogen using Vitrobot Mark IV (Thermo Fisher Scientific). Before data acquisition, the sample quality was evaluated by a 200 kV Talos Arctica G2 electron microscope (FEI). The well-prepared grids were selected for data acquisition by using a 300 kV Titan Krios G3 electron microscope (FEI) equipped with a K3 Summit direct electron detector (Gatan) at a nominal magnification of 81,000× and a GIF-Quantum LS Imaging energy filter with a slit width of 20 eV. Images were collected with a physical pixel size of 1.071 Å and a defocus ranging from –0.8 μm to –1.5 μm by SerialEM.^42^ Each image stack comprised 40 frames in a total of 3 s with 0.075 s exposure per frame, and the total dose was 70 electrons per Å^2^.

### Cryo-EM data processing and map construction

The image stacks were subjected to beam-induced motion correction by MotionCor2.^43^ Contrast transfer function (CTF) parameters for each image were determined by Ctffind4.^44^ Particle picking, two-dimensional (2D) classification, three-dimensional (3D) classification, non-uniform refinement, local refinement and local resolution estimation were performed by cryoSPARC^45,46^ except otherwise noted. Topaz^47,48^ was used for particle re-picking. Bayesian polishing and CTF refinement were performed by RELION3.1.^49^ The resolutions of density maps were calculated by the gold-standard Fourier shell correlation (FSC) with the 0.143 criterion.

For the mGlu2–mGlu3 heterodimer in the presence of LY341495 alone, a total of 24,931 movies were collected from three independent experiments and processed separately as four datasets of 5696, 8785, 5788, and 4662 movies. All datasets were submitted to beam-induced motion correction and CTF determination. A total of 2,852,718, 4,113,981, 4,646,955, and 2,912,582 particle projections were located and extracted by reference-free auto-picking and subjected to several rounds of 2D classification to discard false-positive particles. Four to six initial models generated by cryoSPARC were used as reference models for 3D classification. The best-looking classifications were marked as training data for Topaz to re-pick particles in each dataset. The re-picked 955,795, 942,588, 830,436, and 1,498,002 particles were subjected for several rounds of 3D classification and then a total of 1,096,051 particles were selected from the four datasets and combined for the last round of 3D classification. Finally, the best-looking datasets of 512,450 and 199,444 particles were subjected to non-uniform refinement, resulting in two maps with global resolutions at 2.8 Å and 3.4 Å, which enabled determination of two inactive structures in dimerization modes I and II, respectively.

For the mGlu2–mGlu3 heterodimer in the presence of LY341495 and NAM563, a total of 22,122 movies were collected from three independent experiments and processed separately as three datasets of 4078, 6559, and 11,485 movies. After beam-induced motion correction and CTF determination, a total of 3,079,661, 5,107,167, and 5,629,479 particle projections were located and extracted by reference-free auto-picking and subjected to several rounds of 2D classification to discard false-positive particles. Four to eight initial models generated by cryoSPARC were used as reference models for 3D classification. After several rounds of 3D classification, two datasets of 884,538 and 1,853,997 particles were subjected to further 3D classification. Finally, the best-looking classes of 516,615 and 590,649 particles were subjected to non-uniform refinement, resulting in two maps both with global resolution at 3.0 Å, which enabled determination of two inactive structures in dimerization modes I and II.

For the mGlu2–mGlu3 heterodimer in the presence of LY341495, NAM563, and LY2389575, a total of 24,646 movies were collected from three independent experiments and processed separately as four datasets of 4986, 5026, 9296, and 5338 movies. Similarly, after motion correction and CTF determination, a total of 2,096,341, 3,620,673, 4,283,519, and 3,601,874 particle projections were respectively produced and subjected to several rounds of 2D classification and 3D classification with four to six initial models. After several rounds of 3D classification, 1,988,255 particles were selected for the last round of 3D classification. Finally, the best-looking classes of 1,162,068 and 749,008 particles were submitted to non-uniform refinement, resulting in two maps with global resolutions at 2.9 Å and 3.0 Å, which enabled determination of two inactive structures in dimerization modes I and III, respectively.

To exclude existence of dimerization mode III in the datasets of mGlu2–mGlu3 in the presence of LY341495 alone or LY341495 together with NAM563 as well as existence of dimerization mode II in the dataset of mGlu2–mGlu3 in the presence of LY341495, NAM563, and LY2389575, we performed another round of heterogeneous refinement by a selected reference model of the specific inactive state together with five to seven initial models generated from ab-initio reconstruction. The classifications from heterogeneous refinement were then subjected to non-uniform refinement for further checking of the TMD dimer interface. For each dataset, we first tested particles directly from the last round of 3D classification to confirm that no reference model-induced error of classification occurred for the previously determined models. Then, more particles from earlier 3D classification were merged in order to search for other inactive state and tested in the same strategy. The results showed that dimerization mode III was not found in the datasets of mGlu2–mGlu3 in the presence of LY341495 alone or LY341495 together with NAM563. The particles classified by the reference model of dimerization mode III belong to dimerization mode II after reconstruction. Furthermore, dimerization mode II was also not found in the dataset of mGlu2–mGlu3 in the presence of LY341495, NAM563, and LY2389575. The particles classified by the reference model of dimerization mode II were finally reconstructed into dimerization mode III or failed to generate a model because of insufficient particles from classification.

For the mGlu2–mGlu3 heterodimer in the presence of NAM563 alone, a total of 15,261 movies were collected from two independent experiments and processed separately as three datasets of 6668, 3568, and 5025 movies. After beam-induced motion correction and CTF determination, a total of 2,173,357, 1,636,605, and 1,803,145 particle projections were respectively extracted by reference-free auto-picking and subjected to several rounds of 2D classification to discard false-positive particles. The previously generated mGlu2–mGlu7 density map^16^ was used as the reference model for 3D classification by RELION, and the best class in each iteration was used as the reference model for the next round of 3D classification. After several rounds of 3D classification, 839,474 particles were selected for the last round of 3D classification by cryoSPARC. Four initial models generated by cryoSPARC were used as reference models for 3D classification. Finally, the best-looking classification of 460,025 particles was subjected to non-uniform refinement, resulting in a map with global resolution at 3.3 Å. Furthermore, 207,479 particles from the second-best class were also refined, yielding a map at overall resolution of 3.4 Å, which was discarded due to poor densities at the TMDs. In addition, 1,284,319 particles from the previously discarded classifications were double-checked for alternative conformations. After three rounds of 2D classification, 277,407 particles were selected for another round of 3D classification. The best-looking class of 130,811 particles was subjected to non-uniform refinement, yielding a map with global resolution at 3.7 Å. However, this map was also discarded due to poor densities at the TMDs.

For the mGlu2–mGlu3 heterodimer in the presence of glutamate, JNJ-40411813, and CaCl_2_, a total of 25,416 movies were collected from three independent experiments and processed separately as three datasets of 5746, 8678, and 10,992 movies. After beam-induced motion correction and CTF determination, a total of 5,226,045, 7,805,613, and 10,826,306 particle projections were respectively produced and subjected to several rounds of 2D and 3D classifications. After several rounds of 3D classification, a total of 2,648,248 particles from the three datasets were selected and combined for another round of 3D classification. We found that two groups of particles are of different conformations. So we further subjected the group with 1,460,428 particles to Bayesian polishing and 3D classification focused on the TMDs in RELION, and another group with 426,259 particles to 3D classification in cryoSPARC with two initial models generated by cryoSPARC as reference models. Finally, the best-looking classes of 890,025 and 252,188 particles were subjected to non-uniform refinement, resulting in two maps with global resolutions at 2.8 Å and 3.7 Å, respectively.

For the G_i1_-bound mGlu2–mGlu3 in the presence of glutamate, JNJ-40411813, and CaCl_2_, a total of 21,278 movies were collected from three independent experiments and processed separately as three datasets of 4600, 11,812, and 4866 movies. After beam-induced motion correction and CTF determination, a total of 5,495,273, 13,358,582, and 4,170,113 particle projections were respectively located and extracted by reference-free auto-picking and subjected to several rounds of 2D classification to discard false-positive particles. Four to six initial models generated by cryoSPARC were used as reference models for 3D classification. After several rounds of 2D classification and 3D classification, a total of 1,621,521 particles from the three datasets were selected and combined for the last round of 3D classification. Finally, after Bayesian polishing and CTF refinement, the best-looking class of 994,275 particles were subjected to non-uniform refinement, resulting in a map with global resolution at 3.3 Å.

For the G_i1_-bound mGlu2–mGlu4 in the presence of glutamate, JNJ-40411813, and ADX88178, a total of 32,979 movies were collected from three independent experiments and processed separately as two datasets of 11,524 and 21,455 movies. Similarly, after beam-induced motion correction and CTF determination, a total of 5,787,063 and 9,576,500 particle projections were respectively produced by reference-free auto-picking and subjected to several rounds of 2D classification to discard false-positive particles. Four initial models generated by cryoSPARC were used as reference models for 3D classification. In these two datasets, two major classifications were observed, including G_i1_-bound and G_i1_-free mGlu2–mGlu4 heterodimers. The particles of the G_i1_-bound mGlu2–mGlu4 classifications from both datasets were submitted to Topaz as training data for particle re-picking. 2,974,846 and 3,661,135 particles were re-extracted for further 2D classification and 3D classification. Finally, 939,819 particles of the G_i1_-bound mGlu2–mGlu4 classifications were polished and subjected to non-uniform refinement, resulting in a map with global resolution at 3.6 Å. Local refinements focused on the ECDs and TMDs–G_i1_ generated two maps with resolutions at 2.9 Å and 3.4 Å, respectively. The two maps were then merged by ‘Combine Focused Maps’ in Phenix1.20^50^ as a composite map for model building. In addition, 653,804 particles of the G_i_-free mGlu2–mGlu4, which were from the first dataset, were also subjected to non-uniform refinement, resulting in a map with global resolution at 2.9 Å.

For the mGlu4–G_i3_ complex from our previous study,^15^ a well-processed dataset of 3,446,039 particles was selected and submitted to two rounds of 2D classification to discard false-positive particles. Two rounds of 3D classification were then performed with six and four initial models generated by cryoSPARC. The best-looking classes of 839,200 particles were subjected to non-uniform refinement, resulting in a map with a global resolution at 3.4 Å. Subsequently, local refinements focused on the ECDs and TMDs–G_i3_ generated two maps with resolutions at 2.9 Å and 3.3 Å, respectively, which were then submitted to Phenix1.20 to generate a composite map for modeling. Processing workflows of all the above cryo-EM data are shown in Supplementary information, Figs. S2 and S3.

### Model building and refinement

A model of the mGlu2 subunit was built by rigid-body fitting using the previously published mGlu2 homodimer structures^15,16^ (PDB IDs: 7E9G for the G_i1_-bound mGlu2–mGlu3 and mGlu2–mGlu4; 7EPB for the intermediate active heterodimers; 7EPA for the inactive mGlu2–mGlu3 structures). A model of the mGlu3 subunit in the active state was generated based on the mGlu2 homodimer structure (PDB ID: 7E9G) using SWISS-MODEL server,^51^ For the inactive state of mGlu3, the model was built by merging the crystal structure of the inactive mGlu3 VFT (PDB ID: 5CNM) and the CRD and TMD in the active model, which was followed by the rigid-body fitting. A model of the mGlu4 subunit was built using the published mGlu4 homodimer structure^15^ (PDB ID: 7E9H). The G_i1_ protein model was derived from the structure of FPR1-G_i1_ complex^52^ (PDB ID: 7WVU) and the scFv16 model was from the structure of scFv16-G_i_ complex (PDB ID: 6CRK). Ligand coordinates and geometry restraints were generated using phenix.elbow.^53^ All the models were initially docked into the maps in UCSF ChimeraX,^54^ and were subjected to Coot^55^ for manual refinement and to Phenix for real-space refinement.^53^ The final models were validated by MolProbity^56^ and molecular graphics were prepared by PyMOL (https://pymol.org/2/) and UCSF ChimeraX. The data processing and refinement statistics are provided in Supplementary information, Table S1.

### BRET assay using TRUPATH biosensors

To monitor the G_i_ protein activation of the mGlu2–mGlu3 and mGlu2–mGlu4 heterodimers, a BRET assay using TRUPATH biosensors was conducted to detect the interaction between the Gα and Gγ subunits.^57^ The Flag-tagged wild-type and mutant receptor genes, the C-termini of which were substituted by the C1 or C2 tail of GABA_B_ (mGlu2–C1, mGlu3–C2, and mGlu4–C2) to allow only heterodimer to reach cell surface,^58^ were cloned into the pTT5 vector. The HEK293F cells at a density of 1.5 × 10^6^ cells/mL were co-transfected with plasmids of mGlu2, mGlu3 (or mGlu4), Gα-RLuc8, Gβ, Gγ-GFP2 (Addgene kit no. 1000000163) and the glutamate transporter EAAT1 at a ratio of 5:5:2:2:2:4 (final plasmid concentration, 1 μg/mL) and cultivated in 5% CO_2_ at 37 °C for 48 h.

The cells were plated into 96-well white plates at a density of 40,000 cells per well, mixed with 60 μL of assay buffer containing 1× Hank’s balanced salt solution (HBSS, Thermo Fisher Scientific) and 20 mM HEPES, pH 7.4, and incubated at 37 °C for 60 min. 10 μL of freshly prepared 50 μM coelenterazine 400a (Nanolight Technologies) was then added, and the samples were equilibrated at room temperature for 5 min. Then, the fluorescence was measured by Synergy II (Bio-Tek) plate reader with 410-nm (RLuc8–coelenterazine 400a) and 515-nm (GFP2) emission filters for 5 min. The PAMs were dissolved in dimethyl sulfoxide (DMSO) (JNJ-40411813 at a concentration of 10 mM; ADX88178 at a concentration of 50 mM) and glutamate was dissolved in ddH_2_O at pH 7.4 to a concentration of 100 mM as stock solutions. The ligands were diluted to different concentrations with assay buffer upon assay. The cells were then treated with 30 μL ligand for 5 min followed by serial measurements for 5 times. The last measurements were used in all analyses. The BRET ratios were calculated as the ratio of the GFP2 emission to RLuc8 emission. Statistics were normalized to the wild-type response and figures were prepared by Prism 9 (GraphPad Software, LLC).

### Inositol phosphate (IP) accumulation assay

To measure the basal activity of mGlu2–mGlu3, IP accumulation assay was performed by using IP-One G_q_ assay kit (PerkinElmer) following the manufacturer’s instructions. The wild-type and mutant *mGlu2* and *mGlu3* genes, which contain the GABA_B_ tails, were cloned into the pTT5 vector. The HEK293F cells were co-transfected with the plasmids of mGlu2, mGlu3, the chimeric Gα protein Gα_qi9_^59^ and EAAT1 at a ratio of 1:1:2:1 with the final concentration of 1 μg/mL. The heterodimers were expressed following the same protocol of the BRET assay. After 48 h, surface expression levels were measured with the monoclonal anti-Flag M2-FITC antibody (Sigma; 1:100 diluted in TBS + 4% BSA) using a flow cytometry reader (Millipore). The cells were then loaded into 384-well white plates (40,000 cells per well) and incubated with stimulation buffer (provided by manufacturer) at 37 °C for 90 min. The basal activity of mGlu2–mGlu3 was calculated by subtracting the IP production measured in the control (cells co-transfected with Gα_qi9_, EAAT1, and the empty pTT5 vector) for the wild-type heterodimer and all the mutants.

### Fluorescence-labeled cysteine crosslinking assay

The crosslinking assay of mGlu2–mGlu3 was performed as previously described.^60^ The N-terminally SNAP-tagged, wild-type and mutant mGlu2 and mGlu3, with their C-termini replaced by the coiled-coil regions C1 and C2 of GABA_B_, respectively, were cloned into the pcDNA3.1 vector and co-expressed in HEK293T cells (obtained from Cell Bank at the Chinese Academy of Sciences; cells were routinely tested for mycoplasma contamination). The cells were co-transfected with the plasmids of mGlu2, mGlu3, and EAAT1 at a ratio of 2:2:1 with the final concentration of 1 μg/mL. After 48 h, the cells were plated into 6-well plates (800,000 cells per well) and cultured in 5% CO_2_ at 37 °C for 24 h. The cells were then labeled with 100 nM SNAP-Surface 649 (NEB) at 37 °C for 1 h. After discarding the supernatant, the cells were incubated with PBS in the absence or presence of different ligands (30 μM LY341495, 10 μM NAM563 or 10 μM LY2389575) at 37 °C for 30 min. The cells were further incubated with crosslinking buffer (16.7 mM Tris, pH 8.0, 100 mM NaCl, 1 mM CaCl_2_, and 5 mM MgCl_2_) supplemented with or without 1.5 mM CuP at room temperature for 15 min. The reaction was terminated by adding 10 mM *N*-ethylmaleimide and incubating at 4 °C for 15 min. The cells were collected by centrifugation at 14,000× *g* for 30 min, and lysed with CelLytic M (Sigma) at 4 °C for 1 h. The supernatant was collected by centrifugation at 14,000× *g* at 4 °C for 30 min, and 15 μL of the supernatant was mixed with 5 μL SDS loading buffer and loaded to 5% SDS-PAGE. After electrophoresis, the proteins in the PAGE were transferred into polyvinylidene difluoride membranes (Sigma). The membranes were imaged by ChemiDoc MP Imaging System (Bio-Rad).

### MD simulations

The conformational rearrangement of glutamate-bound mGlu2–mGlu3 VFT heterodimer was simulated by using GaMD, a sophisticated enhanced sampling MD method that adds a boost potential to smoothen the biomolecular potential energy surface which allows for the quantitative measurement of the structural motions of biomolecules with endurable computational time.^33,34^ The atomic coordinates of the mGlu2–mGlu3 VFTs were retrieved from the cryo-EM structure of the inactive mGlu2–mGlu3 with both VFTs adopting open conformations and the antagonists removed. Two glutamate molecules that attach to the top lobes of the two subunits were modeled based on the glutamate-binding mode in the mGlu2 subunit in the Rco structure of the mGlu2–mGlu3 heterodimer that was purified in the presence of glutamate, JNJ-40411813, and CaCl_2_. The missing loops (S111–D131 in mGlu2 and K119–I134 in mGlu3) were reconstructed and refined using the cyclic coordinate descent (CCD) and kinematic closure (KIC) protocols in Rosetta v3.10, respectively.^61,62^ The protonation states of all titratable residues at pH 7.5 were evaluated using Schrodinger suite software. All residues were found in their standard protonation states. After a detailed inspection of the environment surrounding each histidine residue, all histidines were neutral except H41 in mGlu2, which was positively charged. However, the residues H56, H97, and H390 in mGlu2 and H369, H376, and H498 in mGlu3 were protonated on Nδ position, while the remaining histidines on Nε. S–S linkages were detected between C50–C92, C355–C362, C400–C407 in mGlu2, and C57–C99, C361–C373, C412–C419 in mGlu3.

The protein system was solvated in a cubic box with a buffer region of at least 11 Å from any protein atom to the limits of the box, in which a total of 39,798 water molecules were filled and Na^+^/Cl^–^ ions were added to neutralize the protein charges. AMBER 18 suite of program^63^ was employed for simulations with the underlying force fields of FF14SB force field^64^ for protein, generalized AMBER force field (GAFF)^65^ for isolated glutamate, and TIP3P model^66^ for water molecules.

The constructed system was first minimized for 50,000 steps and then heated to 300 K, with the heavy atoms of the protein and the bound glutamate molecules being fixed using a harmonic restraint (the force constant is 10.0 kcal·mol^–1^·Å^–2^). Subsequently, the protein was relaxed by two steps of equilibrium at constant temperature of 300 K and constant pressure of 1 atm (*NPT* ensemble): 2 ns for relaxing protein side chain and 2 ns for protein main chain. The SHAKE algorithm was used to fix all covalent bonds involving hydrogen atoms and periodic boundary conditions were used to avoid edge effects.^67^ The Particle Mesh Ewald method was applied to treat long-range electrostatic interactions and the cutoff distance for long-range terms (electrostatic and vdW energies) was set as 10.0 Å.^68^ The Langevin dynamics with a collision frequency of 3.0 ps^−1^ was adopted to control the temperature. Finally, the GaMD simulations were performed on the equilibrated system using the GaMD module implemented in the GPU version of AMBER 18, including a 10-ns short conventional MD simulation for collecting the potential statistics to define GaMD acceleration parameter values, a 10-ns equilibration after adding the boost potential, and finally 4 independent production simulations with randomized initial atomic velocities, each lasting 1–2 μs.

All GaMD simulations were run at the ‘dual-boost’ level by setting the reference energy to the lower bound, with one boost potential being applied to the total potential and the other to the dihedral energetic term. The average and the standard deviation (SD) of the system potential energies were calculated every 500,000 steps (1.0 ns). The upper limit of the boost potential SD was set to 6.0 kcal/mol for both dihedral and the total potential energetic terms. The coordinates were saved every 10,000 steps. The free energy landscape (FEL) spanned by specific collective variables was calculated using the accompanied reweighting algorithm of GaMD.

Additionally, a conventional MD simulation was performed to evaluate the intermolecular energies in the open state of the glutamate-bound mGlu2–mGlu3 VFTs, lasting ~200 ns at constant temperature of 300 K and constant pressure of 1 atm. The starting system structure used in the conventional MD simulation is the same as that in GaMD simulation.

### Supplementary information

Supplementary information, Figure S1

Supplementary information, Figure S2

Supplementary information, Figure S3

Supplementary information, Figure S4

Supplementary information, Figure S5

Supplementary information, Figure S6

Supplementary information, Figure S7

Supplementary information, Figure S8

Supplementary information, Table S1

Supplementary information, Table S2

## Data Availability

Atomic coordinates and cryo-EM density maps for the six structures of the inactive mGlu2–mGlu3 have been deposited in the Protein Data Bank (PDB) under identification codes 8JCU, 8JCV, 8JCW, 8JCX, 8JCY, and 8JCZ, and in the Electron Microscopy Data Bank (EMDB) under accession codes EMD-36165, EMD-36166, EMD-36167, EMD-36168, EMD-36169, and EMD-36170, respectively. Atomic coordinates and cryo-EM density maps for the two mGlu2–mGlu3 structures in the Rco conformational states have been deposited in the PDB under identification codes 8JD0 and 8JD1, and in the EMDB under accession codes EMD-36171 and EMD-36172, respectively. Atomic coordinates and cryo-EM density maps for the G_i1_-free and G_i1_-bound structures of active mGlu2–mGlu3 and the G_i1_-free and G_i1_-bound structures of active mGlu2–mGlu4 have been deposited in the PDB under identification codes 8JD2, 8JD3, 8JD4, and 8JD5, respectively, and in the EMDB under accession codes EMD-36173, EMD-36174, EMD-36175, and EMD-36176, respectively. Atomic coordinates and cryo-EM density maps for the newly updated mGlu4–G_i3_ structure has been deposited in the PDB under identification code 8JD6 and in the EMDB under accession code EMD-36177.
