# Supplementary material for: Structural insights into dimerization and activation of the mGlu2–mGlu3 and mGlu2–mGlu4 heterodimers
Source: Cell Res. 2023 Jun 8;33(10):762–74. doi: 10.1038/s41422-023-00830-2 (PMC10543438; doi:10.1038/s41422-023-00830-2)
Supplement: Supplementary file 1 — Supplementary information, Figure S1 [file 41422_2023_830_MOESM1_ESM.pdf]

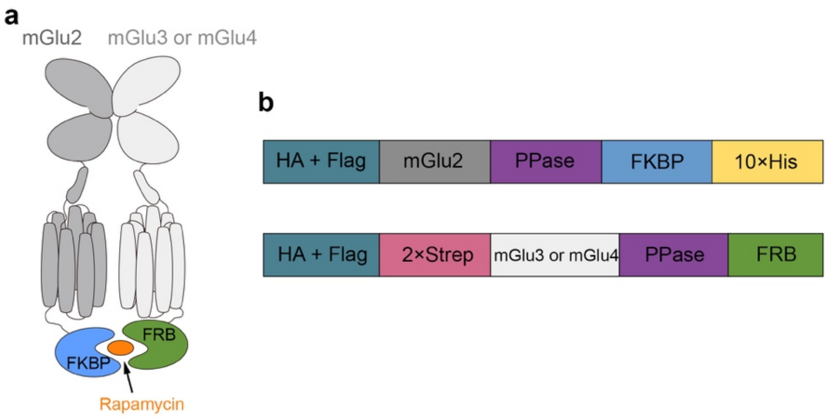

Fig. S1 Construct design of mGlu2, mGlu3, and mGlu4. a Schematic diagram of the heterodimerization. The FKBP and FRB fusion proteins at the C termini of mGlu2 and mGlu3 (or mGlu4), respectively, aid heterodimerization by acting as an interaction pair in the presence of rapamycin. **b** Construct design. PPase, PreScission protease site.
