# Supplementary material for: Structural insights into dimerization and activation of the mGlu2–mGlu3 and mGlu2–mGlu4 heterodimers
Source: Cell Res. 2023 Jun 8;33(10):762–74. doi: 10.1038/s41422-023-00830-2 (PMC10543438; doi:10.1038/s41422-023-00830-2)
Supplement: Supplementary file 2 — Supplementary information, Figure S2 [file 41422_2023_830_MOESM2_ESM.pdf]

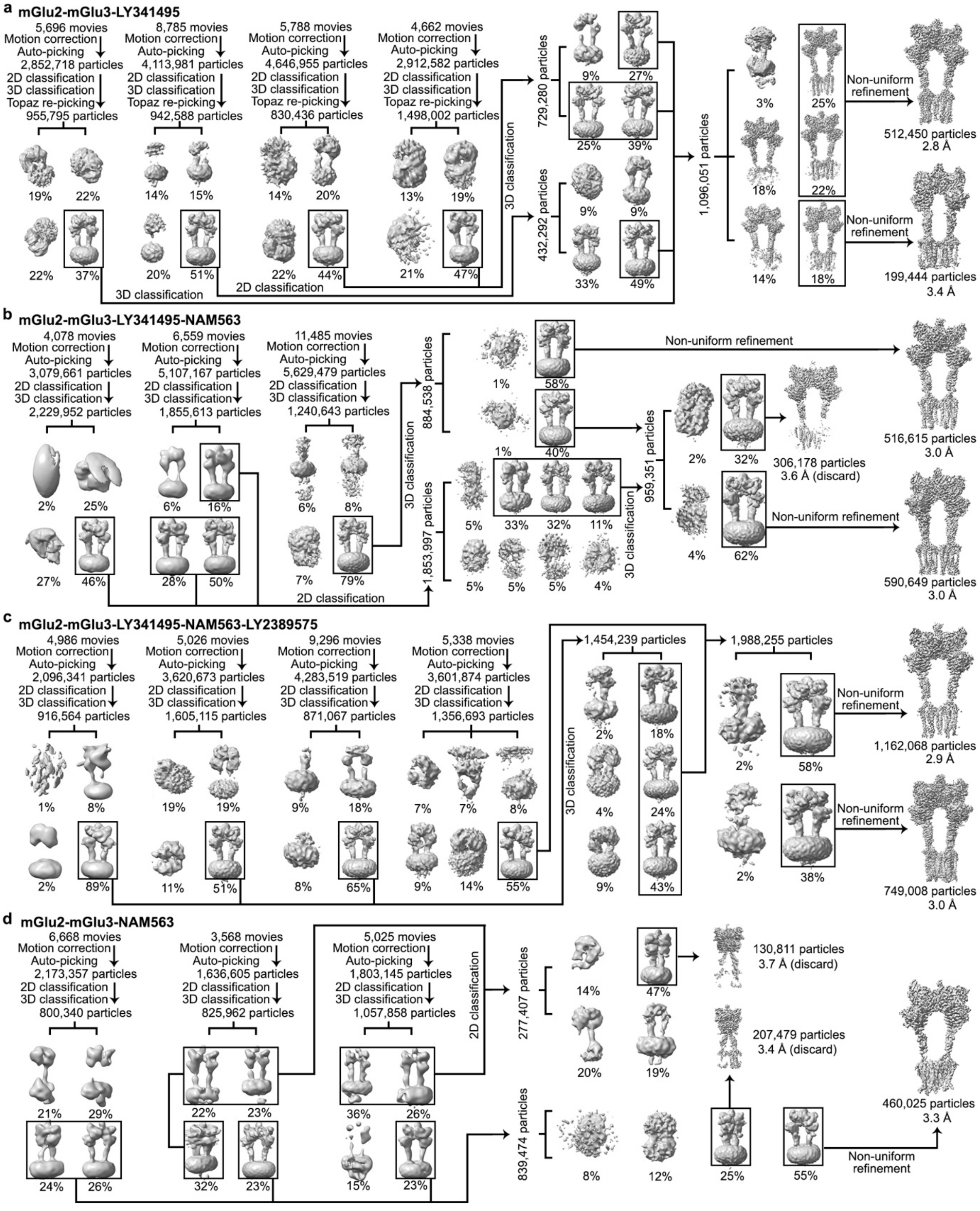

Fig. S2 Cryo-EM workflow of the inactive mGlu2–mGlu3 heterodimers. **a** The mGlu2–mGlu3 heterodimer in the presence of LY341495. **b** The mGlu2–mGlu3 heterodimer in the presence of LY341495 and NAM563. **c** The mGlu2–mGlu3 heterodimer in the presence

of LY341495, NAM563, and LY2389575. **d** The mGlu2–mGlu3 heterodimer in the presence of NAM563.
