# Supplementary material for: Structural insights into dimerization and activation of the mGlu2–mGlu3 and mGlu2–mGlu4 heterodimers
Source: Cell Res. 2023 Jun 8;33(10):762–74. doi: 10.1038/s41422-023-00830-2 (PMC10543438; doi:10.1038/s41422-023-00830-2)
Supplement: Supplementary file 3 — Supplementary information, Figure S3 [file 41422_2023_830_MOESM3_ESM.pdf]

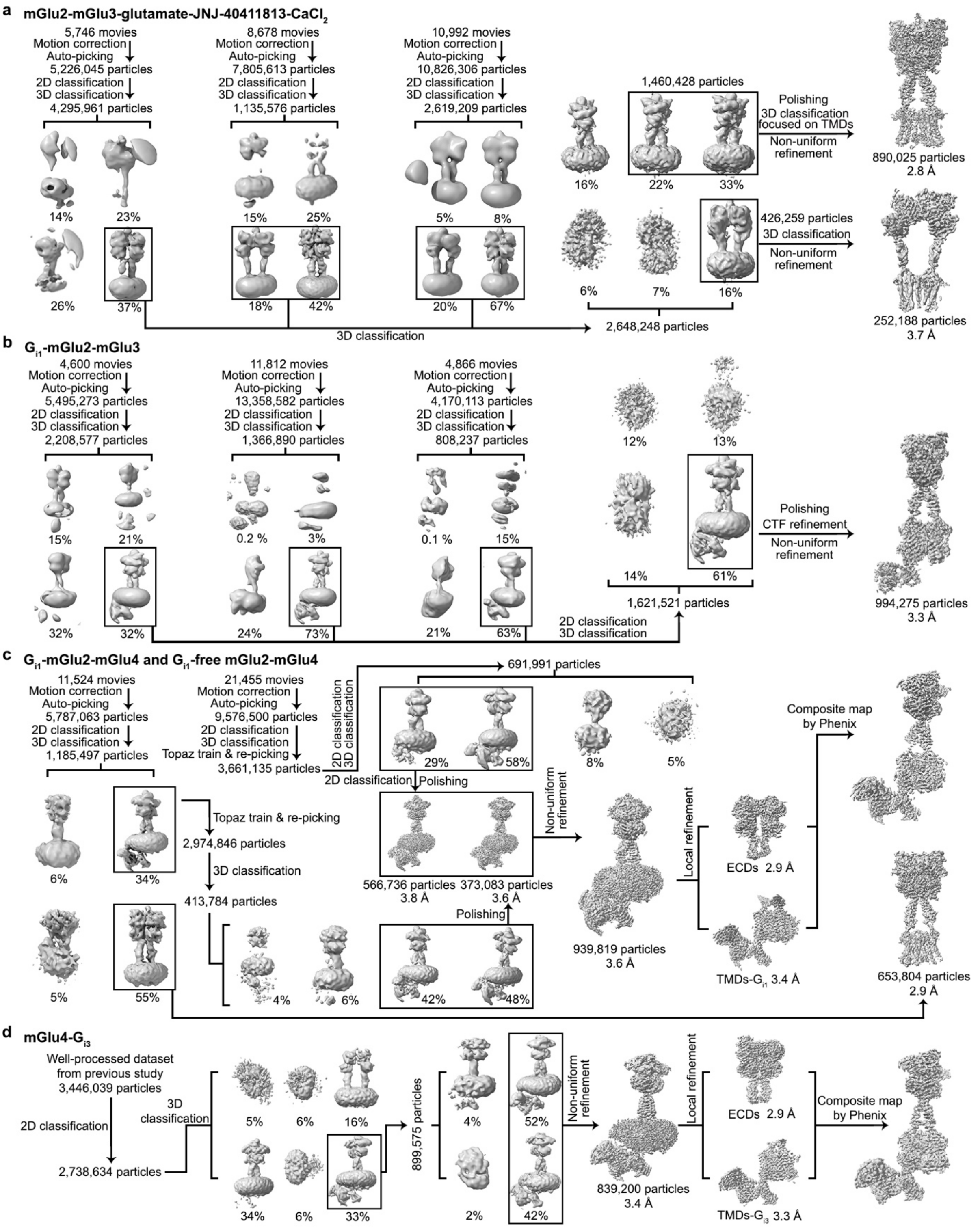


**Fig. S3 Cryo-EM workflow of the agonist-bound mGlu2−mGlu3 and mGlu2−mGlu4 heterodimers and mGlu4 homodimer.** **a** The mGlu2−mGlu3 heterodimer in the presence of glutamate, JNJ-40411813, and $CaCl_2$. **b** The $G_{i1}$-bound mGlu2−mGlu3 heterodimer. **c** The $G_{i1}$-bound and $G_{i1}$-free mGlu2−mGlu4 heterodimers. **d** The $G_{i3}$-bound mGlu4 homodimer.
