# Supplementary material for: Structural insights into dimerization and activation of the mGlu2–mGlu3 and mGlu2–mGlu4 heterodimers
Source: Cell Res. 2023 Jun 8;33(10):762–74. doi: 10.1038/s41422-023-00830-2 (PMC10543438; doi:10.1038/s41422-023-00830-2)
Supplement: Supplementary file 4 — Supplementary information, Figure S4 [file 41422_2023_830_MOESM4_ESM.pdf]

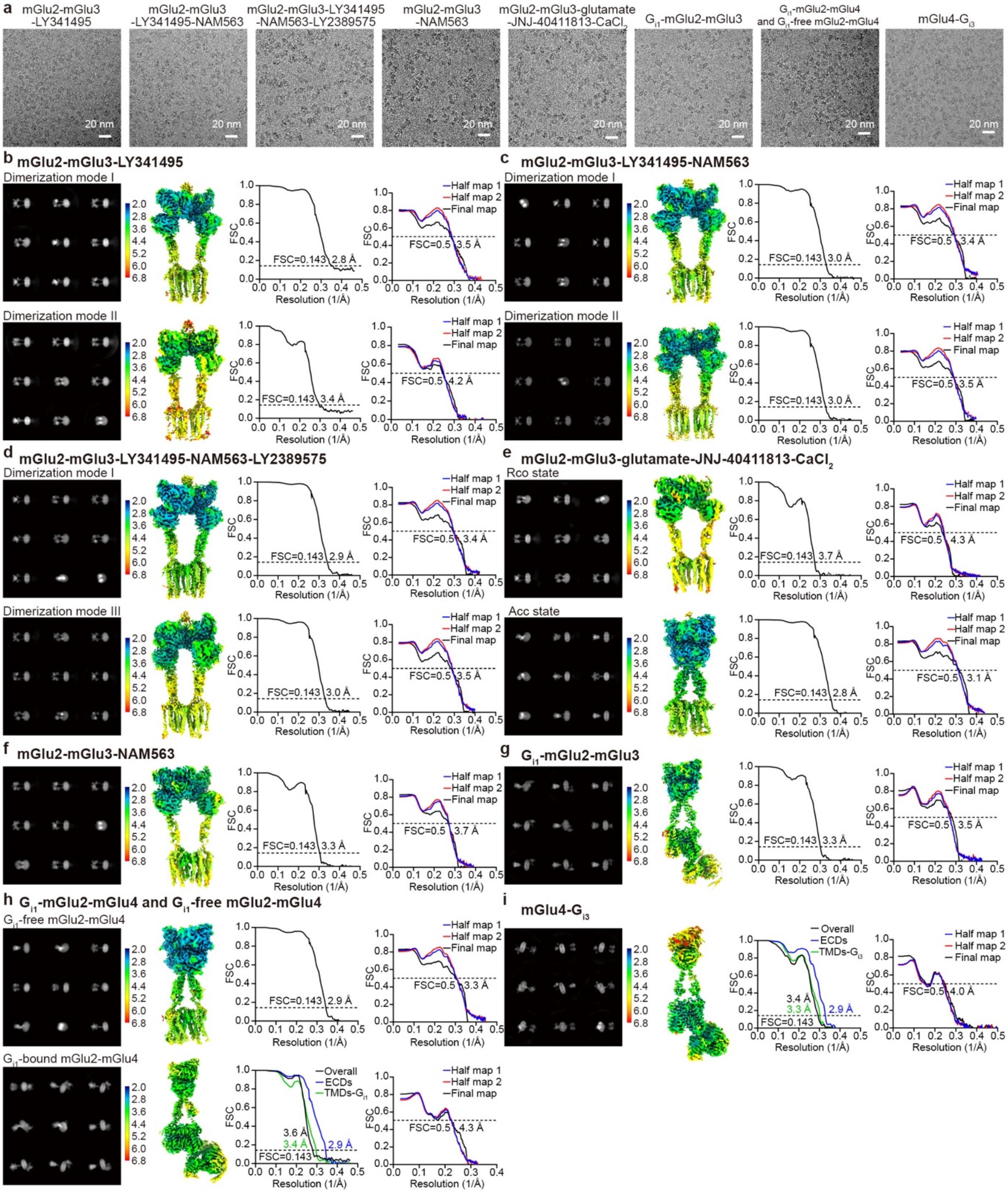

Fig. S4 Cryo-EM processing of the mGlu2–mGlu3 and mGlu2–mGlu4 heterodimers and mGlu4 homodimer. a Representative cryo-EM images from three independent experiments with similar results, except for two independent experiments for the mGlu2–mGlu3–NAM563 complex. **b–i** Processing results, including two-dimensional averages, cryo-EM maps colored according to local resolution (in Å), gold-standard Fourier shell correlation (FSC) curves, and

cross-validations of models to cryo-EM density maps (from left to right). **b** The mGlu2–mGlu3 heterodimer in the presence of LY341495 in dimerization modes I (top) and II (bottom). **c** The mGlu2–mGlu3 heterodimer in the presence of LY341495 and NAM563 in dimerization modes I (top) and II (bottom). **d** The mGlu2–mGlu3 heterodimer in the presence of LY341495, NAM563, and LY2389575 in dimerization modes I (top) and III (bottom). **e** The mGlu2–mGlu3 heterodimer in the presence of glutamate, JNJ-40411813, and $CaCl_2$ in the Rco (top) and Acc (bottom) conformational states. **f** The mGlu2–mGlu3 heterodimer in the presence of NAM563. **g** The G_{i1}-bound mGlu2–mGlu3 heterodimer. **h** The G_{i1}-free (top) and -bound (bottom) mGlu2–mGlu4 heterodimers. **i** The G_{i3}-bound mGlu4 homodimer.
