# Supplementary material for: Structural insights into dimerization and activation of the mGlu2–mGlu3 and mGlu2–mGlu4 heterodimers
Source: Cell Res. 2023 Jun 8;33(10):762–74. doi: 10.1038/s41422-023-00830-2 (PMC10543438; doi:10.1038/s41422-023-00830-2)
Supplement: Supplementary file 5 — Supplementary information, Figure S5 [file 41422_2023_830_MOESM5_ESM.pdf]

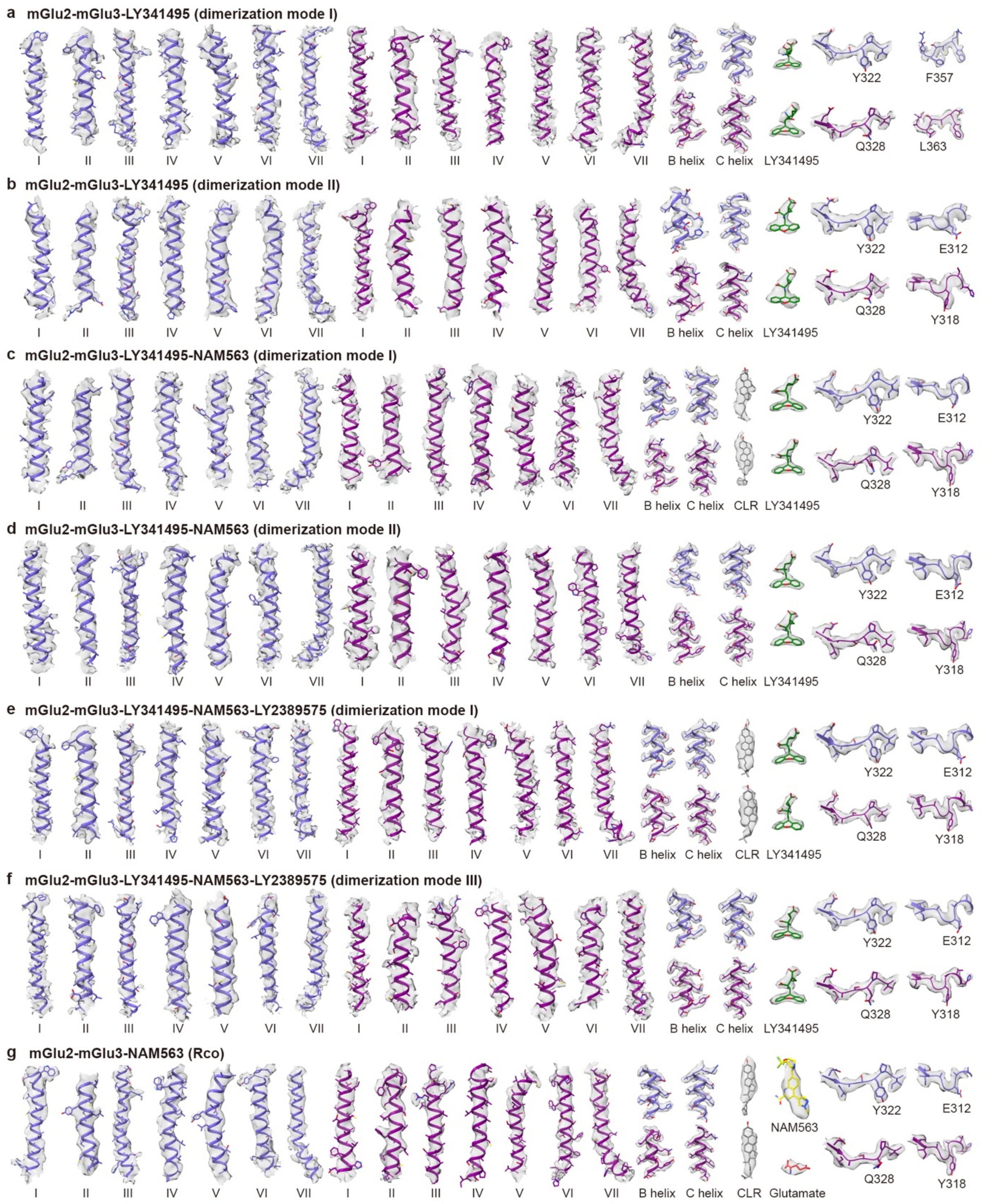

Fig. S5 Cryo-EM density maps of the inactive mGlu2−mGlu3 heterodimers. a, **b** The mGlu2−mGlu3 heterodimer in the presence of LY341495 in dimerization modes I (**a**) and II (**b**). **c**, **d** The mGlu2−mGlu3 heterodimer in the presence of LY341495 and NAM563 in dimerization modes I (**c**) and II (**d**). **e**, **f** The mGlu2−mGlu3 heterodimer in the presence of

LY341495, NAM563, and LY2389575 in dimerization modes I (**e**) and III (**f**). **g** The mGlu2−mGlu3 heterodimer in the presence of NAM563. Maps and models are shown for all transmembrane helices, B and C helices in the VFTs, antagonist (in **a**–**f**), agonist (in **g**), NAM (in **g**), lipid (cholesterol, CLR; in **c**, **e** and **g**) and some non-conserved residues in the two subunits that aided modelling. The models are shown as cartoon and sticks, and colored blue (mGlu2) and purple (mGlu3). The density maps are colored grey.
