# Supplementary material for: Structural insights into dimerization and activation of the mGlu2–mGlu3 and mGlu2–mGlu4 heterodimers
Source: Cell Res. 2023 Jun 8;33(10):762–74. doi: 10.1038/s41422-023-00830-2 (PMC10543438; doi:10.1038/s41422-023-00830-2)
Supplement: Supplementary file 6 — Supplementary information, Figure S6 [file 41422_2023_830_MOESM6_ESM.pdf]

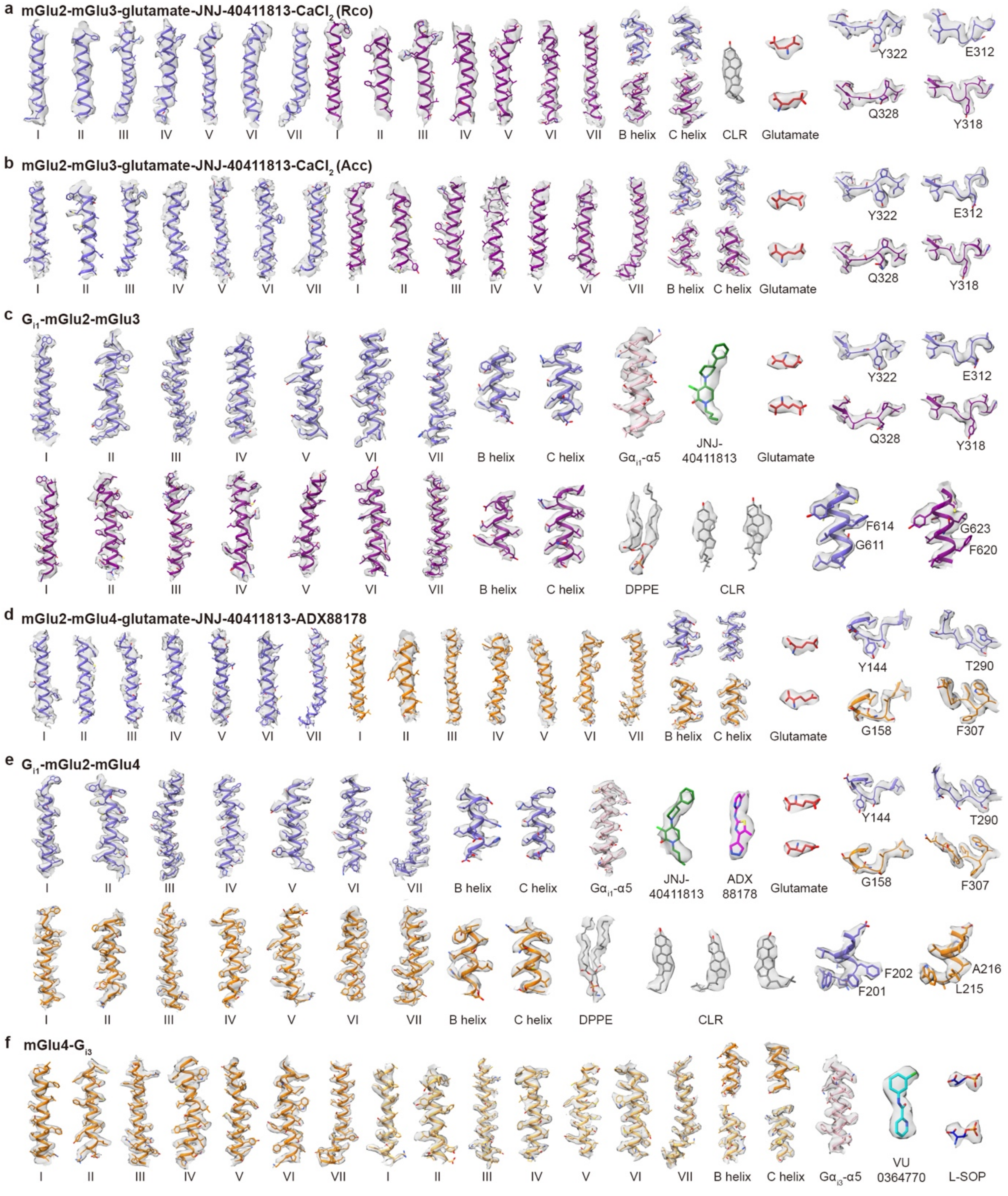

Fig. S6 Cryo-EM density maps of the agonist-bound mGlu hetero- and homodimers. a, **b** The mGlu2–mGlu3 heterodimer in the presence of glutamate, JNJ-40411813, and $CaCl_2$ in the Rco (a) and Acc (b) conformational states. **c** The G_{i1}-bound mGlu2–mGlu3 heterodimer. **d** The mGlu2–mGlu4 heterodimer in the presence of glutamate, JNJ-40411813, and ADX88178. **e** The G_{i1}-bound mGlu2–mGlu4 heterodimer. **f** The G_{i3}-bound mGlu4 homodimer. Maps and

models are shown for all transmembrane helices, B and C helices in the VFTs, agonists, PAMs (in **c**, **e** and **f**), lipids (CLR and DPPE (1,2-dipalmityol-sn-glycero-3-phosphoethanolamine); in **a**, **c** and **d**) and some non-conserved residues in the two subunits that aided modelling. The models are shown as cartoon and sticks, and colored blue (mGlu2), purple (mGlu3), and orange (mGlu4). The density maps are colored grey.
