# Supplementary material for: Structural insights into dimerization and activation of the mGlu2–mGlu3 and mGlu2–mGlu4 heterodimers
Source: Cell Res. 2023 Jun 8;33(10):762–74. doi: 10.1038/s41422-023-00830-2 (PMC10543438; doi:10.1038/s41422-023-00830-2)
Supplement: Supplementary file 7 — Supplementary information, Figure S7 [file 41422_2023_830_MOESM7_ESM.pdf]

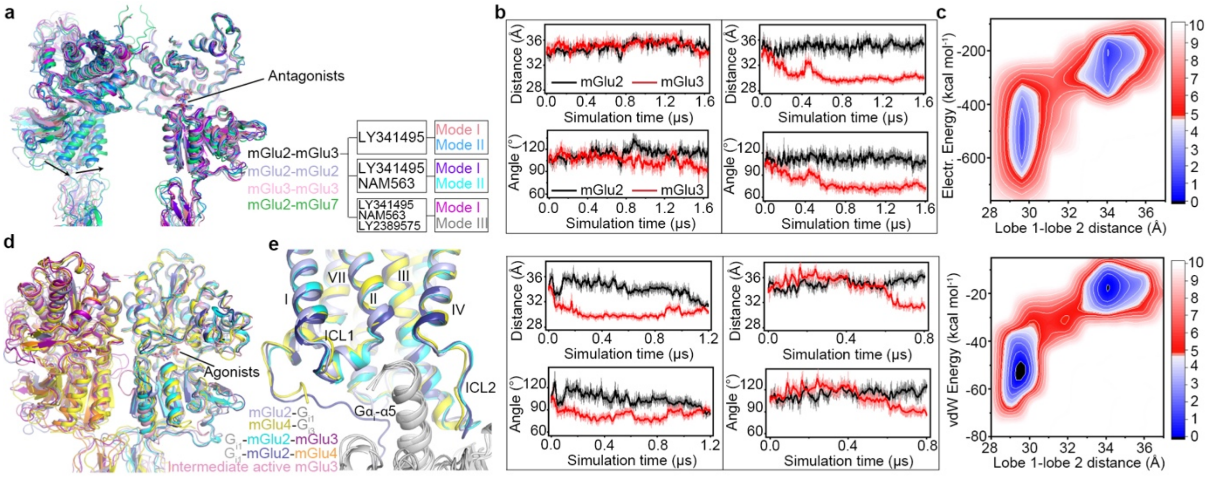

Fig. S7 Structural comparison and GaMD simulations. a Comparison of the inactive VFT conformations in mGlus. The VFTs in the inactive structures of mGlu2–mGlu3 in different dimerization modes, the previously determined structures of the antagonist-bound mGlu2 (PDB ID: 7MTQ) and mGlu3 (PDB ID: 7WI8) homodimers and the inactive mGlu2–mGlu7 heterodimer (PDB ID: 7EPD) are shown in cartoon representation. The antagonists in these structures are shown as sticks. The black arrows indicate differences of the relative positionings of the bottom lobes. **b** Four independent GaMD simulation trajectories of mGlu2–mGlu3 VFT heterodimer as indicated by the time series of the distance between the centroids of lobe 1 and lobe 2 (top) and the lobe 1-lobe 2 subdomain angle (bottom). The trajectories are shown in black (mGlu2) and red (mGlu3). The angle is calculated by projecting the plane of lobe 1 interfacial residues to the plane of lobe 2 interfacial residues. The lobe 1 (top lobe) is the N-terminal subdomain including the residues of K24-T184 and S325-G449 in mGlu2 or R30-T190 and R331-G462 in mGlu3, and the lobe 2 (bottom lobe) includes F189-L319 and Y453-S498 in mGlu2 or D194-L325 and R465-S507 in mGlu3, respectively. **c** Two-dimensional free energy landscapes (FELs) spanned by the distance between the centroids of the lobe 1 and lobe 2 subdomains, and the electrostatic or vdW interaction energy between the lobe 1 and lobe 2 of mGlu3. The contours in the two-dimensional subspace are spaced at intervals of 1.0 kcal mol^{-1}. **d** Comparison of the active VFT conformations in mGlus. The VFTs in the G_i-bound

structures of mGlu2–mGlu3, mGlu2-mGlu4, mGlu2 homodimer (PDB ID: 7E9G), and mGlu4 homodimer, and the structure of the G protein-free, agonist-bound mGlu3 homodimer (PDB ID: 7WIH) are shown in cartoon representation. The agonists in these structures are shown as sticks. **e** G_i-binding site in the mGlu homo- and heterodimers. The G_i-bound subunits and $G\alpha_i$ subunits in the fully active structures of mGlu2–mGlu3, mGlu2–mGlu4, mGlu2, and mGlu4 are shown in cartoon representation.
