# Supplementary material for: Structural insights into dimerization and activation of the mGlu2–mGlu3 and mGlu2–mGlu4 heterodimers
Source: Cell Res. 2023 Jun 8;33(10):762–74. doi: 10.1038/s41422-023-00830-2 (PMC10543438; doi:10.1038/s41422-023-00830-2)
Supplement: Supplementary file 8 — Supplementary information, Figure S8 [file 41422_2023_830_MOESM8_ESM.pdf]

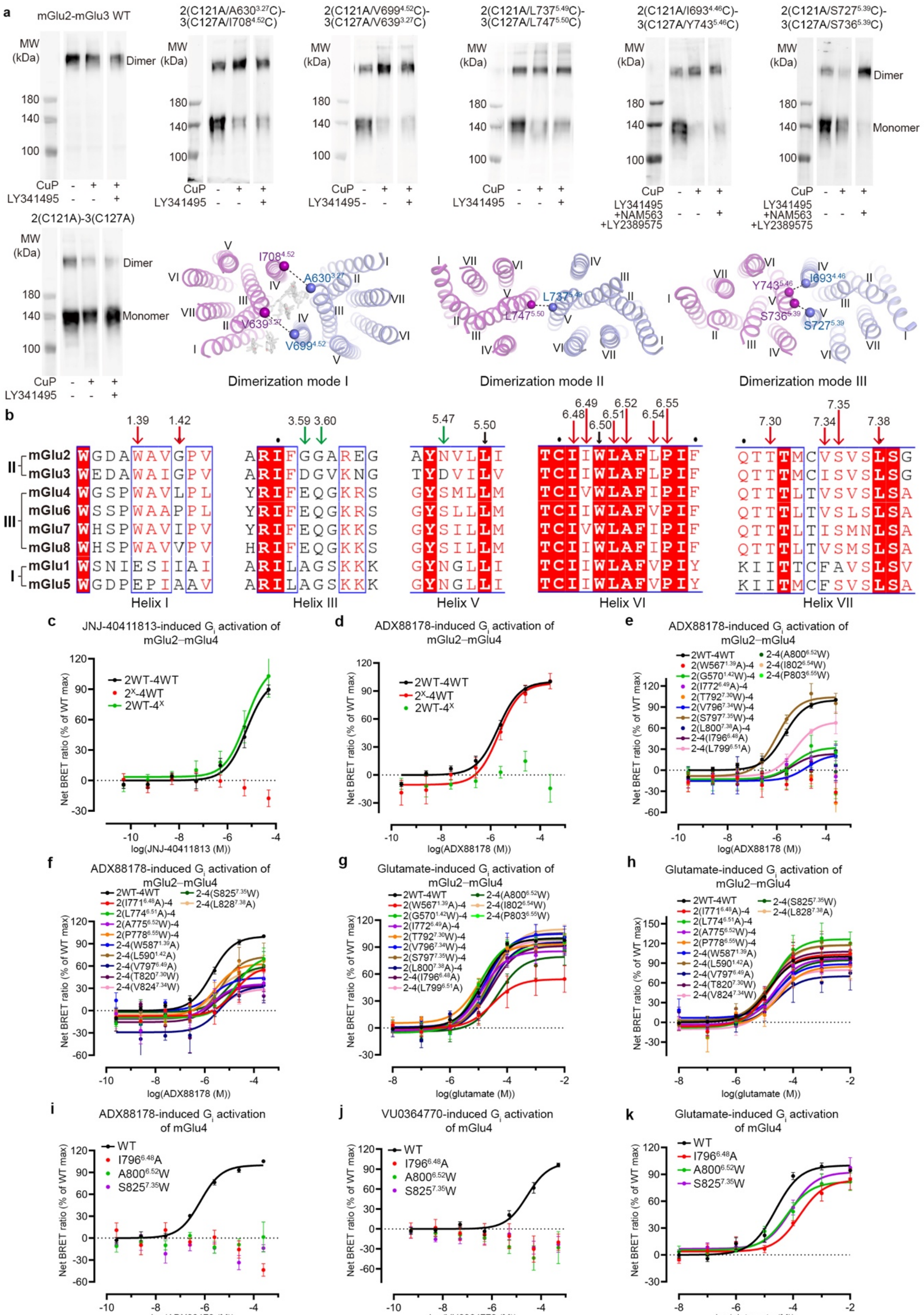
a
mGlu2-mGlu3 WT
Dimer
Monomer
CuP
LY341495
2(C121A)-3(C127A)
Dimerization mode I
Dimerization mode II
Dimerization mode III
b
Helix I
Helix III
Helix V
Helix VI
Helix VII
c
JNJ-40411813-induced G$_i$ activation of mGlu2–mGlu4
d
ADX88178-induced G$_i$ activation of mGlu2–mGlu4
e
ADX88178-induced G$_i$ activation of mGlu2–mGlu4
f
ADX88178-induced G$_i$ activation of mGlu2–mGlu4
g
Glutamate-induced G$_i$ activation of mGlu2–mGlu4
h
Glutamate-induced G$_i$ activation of mGlu2–mGlu4
i
ADX88178-induced G$_i$ activation of mGlu4
j
VU0364770-induced G$_i$ activation of mGlu4
k
Glutamate-induced G$_i$ activation of mGlu4
Net BRET ratio (% of WT max)

Fig. S8 Crosslinking and BRET assays and sequence alignment. a Cysteine crosslinking of the inactive mGlu2–mGlu3 in dimerization modes I-III. The wild-type heterodimer and the mutant mGlu2(C121A)–mGlu3(C127A) were evaluated in parallel as controls. Four independent experiments were performed with similar results. Results from representative experiments are shown. The positions of the cysteine mutations are shown in the inactive structures in dimerization modes I-III at the bottom. **b** Sequence alignment of the TMDs of human mGlus. Colors represent the similarity of residues: red background, identical; red text, strongly similar. The red arrows indicate the positions of residues that interact with the mGlu4 PAM. The green arrows indicate the key positions of 3.59, 3.60 and 5.47 that may determine the G protein-coupling preference of different subunits in the heterodimers. The conserved residues with the modified Ballesteros-Weinstein numbers for class C GPCRs, 5.50 and 6.50, are indicated by black arrows. The alignment was generated using UniProt (http://www.uniprot.org/align/) and the graphic was prepared on the ESPript 3.0 server (http://espript.ibcp.fr/ESPript/cgi-bin/ESPript.cgi). **c**–**k** Agonist- or PAM-induced G_i activation of mGlu2–mGlu3, mGlu2–mGlu4, and mGlu4 measured by the BRET assay. In C and D, the superscript 'X' indicates that the G protein coupling of the subunit was blocked by introducing a mutation in ICL3 (mGlu2, F756S; mGlu3, F765S; mGlu4, F781S). The BRET data are mean ± s.e.m. from at least three independent experiments performed in duplicate. Table S2 provides detailed independent experiment numbers (*n*), statistical evaluation, and expression levels.
