# Supplementary material for: Structural insights into dimerization and activation of the mGlu2–mGlu3 and mGlu2–mGlu4 heterodimers
Source: Cell Res. 2023 Jun 8;33(10):762–74. doi: 10.1038/s41422-023-00830-2 (PMC10543438; doi:10.1038/s41422-023-00830-2)
Supplement: Supplementary file 9 — Supplementary information, Table S1 [file 41422_2023_830_MOESM9_ESM.pdf]

Table S1 Cryo-EM data collection, refinement and validation statistics

	mGlu2–mGlu3–LY341495		mGlu2–mGlu3–LY341495–NAM563		mGlu2–mGlu3–LY341495–NAM563–LY2389575	
**Data collection and processing**						
Magnification	81,000		81,000		81,000	
Voltage (kV)	300		300		300	
Electron exposure (e^{-}/$Å^2$)	70		70		70	
Defocus range (μm)	–0.8 ~ –1.5		–0.8 ~ –1.5		–0.8 ~ –1.5	
Pixel size (Å)	1.071		1.071		1.071	
Symmetry imposed	C1		C1		C1	
Initial particle images (no.)	14,526,236		13,816,307		13,602,407	
	**Mode I**	**Mode II**	**Mode I**	**Mode II**	**Mode I**	**Mode III**
Final particle images (no.)	512,450	199,444	516,615	590,649	1,162,068	749,008
Map resolution (Å)	2.8	3.4	3.0	3.0	2.9	3.0
FSC threshold	0.143	0.143	0.143	0.143	0.143	0.143
Map resolution range (Å)	2.5–6.8	3.2–6.8	2.8–6.8	2.9–6.8	2.8–6.8	2.8–6.8
**Refinement**						
Initial model used (PDB code)	7EPA, 7E9G, 5CNM		7EPA, 7E9G, 5CNM		7EPA, 7E9G, 5CNM	
Model resolution (Å)	3.5	4.2	3.4	3.5	3.4	3.5
FSC threshold	0.5	0.5	0.5	0.5	0.5	0.5
Map sharpening *B* factor ($Å^2$)	–94	–99	–121	–110	–120	–113
Model composition						
Non-hydrogen atoms	10,830	10,667	10,771	10,617	10,874	10,540
Protein residues	1,527	1,514	1,527	1,512	1,525	1,504
Ligands	2	2	2	2	2	2
B factors ($Å^2$)						
Protein	100.90	96.02	65.54	78.28	30.29	47.11
Ligands	51.49	42.39	72.20	36.93	52.31	27.74
R.m.s. deviations						
Bond lengths (Å)	0.002	0.002	0.002	0.003	0.002	0.002
Bond angles (°)	0.502	0.509	0.501	0.454	0.451	0.497
Validation						
MolProbity score	1.56	1.67	1.67	1.61	1.60	1.78
Clashscore	5.20	6.61	5.36	5.05	5.71	8.81
Poor rotamers (%)	0.00	0.00	0.11	0.00	0.00	0.22
Ramachandran plot (%)						
Favored	95.91	95.60	94.46	95.13	95.77	95.56
Allowed	4.09	4.40	5.54	4.87	4.23	4.44
Disallowed	0.00	0.00	0.00	0.00	0.00	0.00

	mGlu2–mGlu3–NAM563	mGlu2–mGlu3–glutamate–JNJ-40411813–$CaCl_2$		G_{i1}–mGlu2–mGlu3	G_{i1}–mGlu2–mGlu4		mGlu4–G_{i3}
**Data collection and processing**							
Magnification	81,000	81,000		81,000	81,000		81,000[a]
Voltage (kV)	300	300		300	300		300[a]
Electron exposure (e^{-}/$Å^2$)	70	70		70	70		70[a]
Defocus range (μm)	–0.8 ~ –1.5	–0.8 ~ –1.5		–0.8 ~ –1.5	–0.8 ~ –1.5		–0.8 ~ –1.5[a]
Pixel size (Å)	1.071	1.071		1.071	1.071		1.045[a]
Symmetry imposed	C1	C1		C1	C1		C1[a]
Initial particle images (no.)	5,613,107	23,857,964		23,023,968	15,363,563		16,656,316[a]
		**Rco state**	**Acc state**		**G_{i1}-free**	**G_{i1}-bound**	
Final particle images (no.)	460,025	252,188	890,025	994,275	653,804	939,819	839,200
Map resolution (Å)	3.3	3.7	2.8	3.3	2.9	2.9 (ECDs), 3.4 (TMDs–G_{i1})	2.9 (ECDs), 3.3 (TMDs–G_{i3})
FSC threshold	0.143	0.143	0.143	0.143	0.143	0.143	0.143
Map resolution range (Å)	3.1–6.8	3.3–6.8	2.7–6.8	3.0–6.8	2.6–6.8	2.8–6.8	2.9–6.8

**Refinement**							
Initial model used (PDB code)	7EPA, 7E9G, 5CNM	7EPA, 7E9G, 5CNM	7EPB, 7E9G	7E9G	7EPB, 7E9H	7E9G, 7E9H	6N51, 6LML[a]
Model resolution (Å)	3.7	4.3	3.1	3.5	3.3	4.3	4.0
FSC threshold	0.5	0.5	0.5	0.5	0.5	0.5	0.5
Map sharpening B factor (Å^2)	–126	–148	–100	–124	–113	–88 (ECDs) –92 (TMDs–G_{i1})	–97 (ECDs) –90 (TMDs–G_{i3})
Model composition							
Non-hydrogen atoms	11,264	10,766	10,730	15,770	10,686	18,037	17,237
Protein residues	1,531	1,506	1,499	2,146	1,492	2,387	2,317
Ligands	2	2	2	3	2	4	3
B factors (Å^2)							
Protein	32.64	50.69	53.03	14.20	82.93	97.40	44.89
Ligands	60.69	88.54	30.75	21.81	48.63	85.59	16.90
R.m.s. deviations							
Bond lengths (Å)	0.002	0.002	0.002	0.002	0.002	0.002	0.003
Bond angles (°)	0.459	0.483	0.426	0.488	0.472	0.491	0.622
Validation							
MolProbity score	1.61	1.80	1.54	1.61	1.55	1.81	1.98
Clashscore	5.89	6.47	4.17	6.09	5.96	7.10	11.31
Poor rotamers (%)	0.10	0.00	0.20	0.00	0.00	0.00	0.17
Ramachandran plot (%)							
Favored	95.79	93.21	95.08	95.96	96.52	93.73	94.25
Allowed	4.21	6.79	4.92	4.04	3.48	6.27	5.75
Disallowed	0.00	0.00	0.00	0.00	0.00	0.00	0.00

[a]Data from previous study.[15]
