# Supplementary material for: Structural insights into dimerization and activation of the mGlu2–mGlu3 and mGlu2–mGlu4 heterodimers
Source: Cell Res. 2023 Jun 8;33(10):762–74. doi: 10.1038/s41422-023-00830-2 (PMC10543438; doi:10.1038/s41422-023-00830-2)
Supplement: Supplementary file 10 — Supplementary information, Table S2 [file 41422_2023_830_MOESM10_ESM.pdf]

Table S2 IP accumulation and G_i activation assays of wild-type and mutant mGlu hetero- and homodimers

Basal activity of mGlu2–mGlu3 measured by IP accumulation assay

	WT/mutants	Basal activity[a]	n[c]	Expression[d]
		% of WT[b]		% of WT[b]
	WT	100	23	100
	WT+LY341495	38 ± 3***	7	/
mGlu2	E604$^{2.37}$W	115 ± 7	7	99 ± 13
	G611$^{2.44}$W	135 ± 6*	7	94 ± 8
	L615$^{2.48}$W	130 ± 5	8	88 ± 10
	A630$^{3.27}$W	159 ± 11***	7	100 ± 14
	T633$^{3.30}$W	162 ± 15***	6	69 ± 11
	L637$^{3.34}$W	135 ± 7*	7	58 ± 6
	S678$^{4.31}$W	143 ± 7*	7	87 ± 18
	A681$^{4.34}$W	135 ± 8*	7	89 ± 20
	L684$^{4.37}$W	144 ± 7**	8	82 ± 8
	S688$^{4.41}$W	150 ± 15**	6	83 ± 17
	V695$^{4.48}$W	109 ± 13	7	78 ± 12
	V699$^{4.52}$W	153 ± 8***	9	82 ± 8
mGlu3	F598$^{1.61}$A	141 ± 16*	7	73 ± 9
	Y616$^{2.40}$A	115 ± 7	7	124 ± 9
	F620$^{2.44}$A	133 ± 10	6	127 ± 19
	L624$^{2.48}$W	142 ± 9*	7	126 ± 11
	C627$^{2.51}$W	149 ± 10**	6	122 ± 14
	F631$^{2.55}$A	139 ± 11*	7	142 ± 11*
	V639$^{3.27}$W	150 ± 13**	5	118 ± 14
	L643$^{3.31}$W	144 ± 11**	7	127 ± 13
	L646$^{3.34}$W	155 ± 11***	8	130 ± 8
	S650$^{3.38}$W	138 ± 4*	7	106 ± 12
	V701$^{4.45}$W	128 ± 7	8	103 ± 15
	S686$^{4.30}$W	131 ± 13	5	114 ± 24
	F690$^{4.34}$A	170 ± 7***	5	147 ± 16
	G694$^{4.38}$W	163 ± 9***	7	100 ± 11

Glutamate-induced G_i activation of mGlu2–mGlu3

WT/mutants[g]	EC_{50} (μM)	EC_{50} ratio[d]	pEC_{50}	E_{max}[f]	n[c]	Expression[d]
			mean ± s.e.m.[b]	% of WT[b]		% of WT[b]
WT	4.4	1	5.36 ± 0.06	100 ± 3	20	100
mGlu2^{X}–mGlu3	17	4	4.76 ± 0.51	19 ± 4***	10	56 ± 5*
mGlu2–mGlu3^{X}	4.5	1	5.34 ± 0.02	97 ± 8	8	97 ± 12
WT+NAM563	19	4	4.72 ± 0.21	90 ± 9	4	100 ± 0
mGlu2^{X}–mGlu3+NAM563	78	18	4.11 ± 0.53	51 ± 15***	4	58 ± 12
mGlu2^{C121A}–mGlu3^{C127A}	2.5	1	5.61 ± 0.27	81 ± 9	8	125 ± 13
mGlu2$^{C121A/I693C}$–mGlu3$^{C127A/Y743C}$	2.8	1	5.56 ± 0.34	44 ± 6***	10	77 ± 22
mGlu2$^{C121A/A726C}$–mGlu3$^{C127A/L709C}$	8.7	2	5.06 ± 0.48	36 ± 8***	5	106 ± 11
mGlu2$^{C121A/V700C}$–mGlu3$^{C127A/S735C}$	6.2	1	5.21 ± 0.44	42 ± 8***	6	112 ± 17
mGlu2$^{C121A/A630C}$–mGlu3$^{C127A/I708C}$	72	16	4.14 ± 1.04	21 ± 12***	3	65 ± 10
WT	13	1	4.90 ± 0.07	100 ± 3	16	100
mGlu2^{YADA}–mGlu3	27	2	4.57 ± 0.20	105 ± 9	5	83 ± 7
mGlu2–mGlu3^{YADA}	122	10	3.91 ± 0.31**	41 ± 6***	4	90 ± 8
mGlu2^{YADA}–mGlu3^{X}	26	2	4.59 ± 0.26	70 ± 8*	4	67 ± 10**

mGlu2^{X}–mGlu3^{YADA}	nd	nd	nd	nd	4	31 ± 4***
mGlu2^{X}–mGlu3	nd	nd	nd	nd	6	78 ± 7*
mGlu2^{X}–mGlu3^{D744N}	22	2	4.66 ± 0.24	84 ± 8	4	76 ± 7*
mGlu2^{X}–mGlu3^{D671G}	224	17	3.65 ± 0.20***	85 ± 8	4	94 ± 8

Glutamate-induced G_i activation of mGlu2–mGlu4

WT/mutants[g]	EC_{50} (μM)	EC_{50} ratio[d]	pEC_{50} mean ± s.e.m.[b]	E_{max}[f] % of WT[b]	n[c]	Expression[d] % of WT[b]
WT	6.3	1	5.20 ± 0.11	100 ± 5	10	100
mGlu2^{X}–mGlu4	11	2	4.96 ± 0.18	102 ± 9	5	41 ± 6**
mGlu2–mGlu4^{X}	nd	nd	nd	nd	5	97 ± 19
WT	13	1	4.88 ± 0.55	100 ± 2	17	100
mGlu2–mGlu4^{X}	42	3	4.38 ± 0.63	13 ± 4***	7	75 ± 9
mGlu2^{G663Q}–mGlu4^{X}	138	10	3.86 ± 0.26*	38 ± 5***	7	82 ± 7
mGlu2^{N735S}–mGlu4^{X}	361	28	3.44 ± 0.47**	33 ± 7***	6	72 ± 8

Glutamate/ADX88178-induecd G_i activation of mGlu2–mGlu4

WT/mutants		ADX88178 EC_{50} (μM)	ADX88178 EC_{50} ratio[d]	ADX88178 pEC_{50} mean ± s.e.m.[b]	ADX88178 E_{max}[f] % of WT[b]	ADX88178 n[c]	Glutamate EC_{50} (μM)	Glutamate EC_{50} ratio[d]	Glutamate pEC_{50} mean ± s.e.m.[b]	Glutamate E_{max}[f] % of WT[b]	Glutamate n[c]	Expression[d] % of WT[b]
WT		2.0	1	5.70 ± 0.07	100 ± 3	18	13	1	4.88 ± 0.55	100 ± 2	17	100
mGlu2	W567$^{1.39}$A	nd	nd	nd	nd	5	31	2	4.51 ± 0.44	55 ± 10***	5	27 ± 5***
mGlu2	G570$^{1.42}$W	4.7	2	5.33 ± 0.46	45 ± 10***	5	10	1	5.00 ± 0.12	105 ± 5	5	81 ± 7
mGlu2	I772$^{6.49}$A	nd	nd	nd	nd	5	13	1	4.89 ± 0.12	88 ± 5	5	39 ± 4*
mGlu2	T792$^{7.30}$W	nd	nd	nd	nd	5	10	1	5.00 ± 0.12	88 ± 5	5	71 ± 8
mGlu2	V796$^{7.34}$W	16	8	4.79 ± 0.74	38 ± 15***	5	19	1	4.73 ± 0.17	104 ± 8	5	84 ± 8
mGlu2	S797$^{7.35}$W	0.99	0.5	6.01 ± 0.19	113 ± 9	5	24	2	4.63 ± 0.13	109 ± 6	5	110 ± 14
mGlu2	L800$^{7.38}$A	nd	nd	nd	nd	4	25	2	4.61 ± 0.20	99 ± 8	4	87 ± 16
mGlu2	I771$^{6.48}$A	17	8	4.78 ± 0.38	64 ± 14	5	14	1	4.85 ± 0.23	110 ± 11	3	132 ± 34
mGlu2	L774$^{6.51}$A	18	9	4.75 ± 0.28	83 ± 13	5	17	1	4.76 ± 0.20	135 ± 12*	3	157 ± 5*
mGlu2	A775$^{6.52}$W	2.4	1	5.63 ± 0.56	47 ± 12	5	22	2	4.66 ± 0.22	101 ± 9	4	106 ± 23
mGlu2	P778$^{6.55}$W	2.6	1	5.59 ± 0.24	70 ± 8	5	32	2	4.50 ± 0.36	90 ± 13	4	64 ± 6
mGlu4	W587$^{1.39}$A	2.2	1	5.66 ± 0.48	44 ± 10*	5	27	2	4.57 ± 0.24	82 ± 8	3	82 ± 40
mGlu4	L590$^{1.42}$A	4.4	2	5.35 ± 0.35	84 ± 14	6	16	1	4.79 ± 0.19	115 ± 9	5	119 ± 21
mGlu4	V797$^{6.49}$A	4.0	2	5.39 ± 0.54	62 ± 16	3	26	2	4.59 ± 0.50	74 ± 15	4	57 ± 18
mGlu4	T820$^{7.30}$W	2.3	1	5.64 ± 0.59	49 ± 13*	4	14	1	4.85 ± 0.25	108 ± 12	4	76 ± 4
mGlu4	V824$^{7.34}$W	6.2	3	5.21 ± 0.67	38 ± 12***	4	20	1	4.71 ± 0.29	91 ± 11	4	74 ± 15
mGlu4	S825$^{7.35}$W	7.8	4	5.11 ± 0.47	62 ± 14*	6	28	2	4.55 ± 0.11	94 ± 4	4	88 ± 17
mGlu4	L828$^{7.38}$A	2.9	1	5.53 ± 0.28	59 ± 8*	5	17	1	4.78 ± 0.21	122 ± 11	4	103 ± 19
mGlu4	I796$^{6.48}$A	4.0	2	5.39 ± 0.54	37 ± 9***	5	29	2	4.54 ± 0.17	91 ± 7	5	113 ± 20
mGlu4	L799$^{6.51}$A	5.5	3	5.26 ± 0.27	78 ± 10	5	16	1	4.78 ± 0.14	102 ± 6	5	107 ± 20
mGlu4	A800$^{6.52}$W	nd	nd	nd	nd	4	48	4	4.32 ± 0.25	84 ± 9	3	87 ± 21
mGlu4	I802$^{6.54}$W	nd	nd	nd	nd	5	32	2	4.49 ± 0.13	112 ± 6	5	170 ± 6**
mGlu4	P803$^{6.55}$W	nd	nd	nd	nd	5	21	2	4.68 ± 0.12	92 ± 5	5	101 ± 13

PAM-induced G_i activation of mGlu2–mGlu4

WT/ Mutants[g]	ADX88178 EC_{50} (μM)	ADX88178 EC_{50} ratio[d]	ADX88178 pEC_{50} mean ± s.e.m.[b]	ADX88178 E_{max}[f] % of WT[b]	ADX88178 n[c]	JNJ-40411813 EC_{50} (μM)	JNJ-40411813 EC_{50} ratio[d]	JNJ-40411813 pEC_{50} mean ± s.e.m.[b]	JNJ-40411813 E_{max}[f] % of WT[b]	JNJ-40411813 n[c]	Expression[d] % of WT[b]
WT	2.0	1	5.70 ± 0.07	100 ± 3	18	6.1	1	5.22 ± 0.16	100 ± 9	9	100
mGlu2^{X}–mGlu4	2.1	1	5.67 ± 0.19	109 ± 10	7	nd	nd	nd	nd	9	54 ± 15*
mGlu2–mGlu4^{X}	nd	nd	nd	nd	7	5.7	1	5.24 ± 0.25	110 ± 17	9	84 ± 12

Glutamate/PAM-induced G_i activation of mGlu4

WT/ mutants	Glutamate EC_{50} (μM)	Glutamate EC_{50} ratio[d]	Glutamate pEC_{50} mean ± s.e.m.[b]	Glutamate E_{max}[f] % of WT[b]	ADX88178 EC_{50} (μM)	ADX88178 EC_{50} ratio[d]	ADX88178 pEC_{50} mean ± s.e.m.[b]	ADX88178 E_{max}[f] % of WT[b]	VU0364770 EC_{50} (μM)	VU0364770 EC_{50} ratio[d]	VU0364770 pEC_{50} mean ± s.e.m.[b]	VU0364770 E_{max}[f] % of WT[b]	n[c]	Expressi-on[d] % of WT[b]

WT	24	1	4.62 ± 0.11	100 ± 4	0.65	1	6.19 ± 0.08	100 ± 3	27	1	4.57 ± 0.13	100 ± 7	7	100
$I796^{6.48}A$	166	7	3.78 ± 0.19*	80 ± 7	nd	nd	nd	nd	nd	nd	nd	nd	5	115 ± 32
$A800^{6.52}W$	57	2	4.24 ± 0.21	76 ± 7	nd	nd	nd	nd	nd	nd	nd	nd	7	29 ± 6*
$S825^{7.35}W$	83	3	4.08 ± 0.19	86 ± 8	nd	nd	nd	nd	nd	nd	nd	nd	6	44 ± 4*

[a]The basal activity was calculated by subtracting the IP production measured in the control ($G\alpha_{qi9}$, EAAT1, and empty PTT5 vector) for the wild-type (WT) heterodimer and all the mutants, and is presented as percent of WT activity.
[b]Data are shown as mean ± s.e.m. from at least three independent experiments. nd (not determined) refers to data where a robust concentration response curve could not be established within the concentration range tested. *P<0.05, **P<0.001, ***P<0.0001 by one-way ANOVA followed by Dunnett's post-test compared to the response of WT.
[c]Sample size, the number of independent experiments performed in technical duplicate.
[d]Protein expression levels of the constructs at the cell surface were determined in parallel by flow cytometry with an anti-Flag antibody (Sigma) and reported as percent compared to the WT from at least three independent measurements performed in duplicate.
[e]The EC_{50} ratio (EC_{50}(mutant)/EC_{50}(WT)) represents the shift between the WT and mutant curves, and characterizes the effect of the mutations on G_i activation.
[f]The maximal response is reported as a percentage of the maximal effect at the WT.
[g]The 'X' indicates that the G protein coupling of the subunit was blocked by introducing a mutation in ICL3 (mGlu2, F756S; mGlu3, F765S; mGlu4, F781S).
